# DNA mechanical flexibility controls DNA potential to activate cGAS-mediated immune surveillance

**DOI:** 10.1038/s41467-022-34858-6

**Published:** 2022-11-19

**Authors:** Lina Wang, Siru Li, Kai Wang, Na Wang, Qiaoling Liu, Zhen Sun, Li Wang, Lulu Wang, Quentin Liu, Chengli Song, Caigang Liu, Qingkai Yang

**Affiliations:** 1 Institute of Cancer Stem Cell, Dalian Medical University, Dalian, 116044 China; 2 CAS Key Laboratory of Separation Sciences for Analytical Chemistry, Dalian Institute of Chemical Physics, Chinese Academy of Sciences, Dalian, China; 3 School of Life Science and Biotechnology, Dalian University of Technology, Dalian, China; 4 Sun Yat-sen University Cancer Center, State Key Laboratory of Oncology in South China, Guangzhou, China; 5 Department of Oncology, Shengjing Hospital of China Medical University, Shenyang, China

**Keywords:** DNA, Enzyme mechanisms, Innate immunity

## Abstract

DNA is well-documented to stimulate immune response. However, the nature of the DNA to activate immune surveillance is less understood. Here, we show that the activation of cyclic GMP-AMP synthase (cGAS) depends on DNA mechanical flexibility, which is controlled by DNA-sequence, -damage and -length. Consistently, DNA-sequence was shown to control cGAS activation. Structural analyses revealed that a conserved cGAS residue (mouse R222 or human R236) contributed to the DNA-flexibility detection. And the residue substitution neutralised the flexibility-controlled DNA-potential to activate cGAS, and relaxed the DNA-length specificity of cGAS. Moreover, low dose radiation was shown to mount cGAS-mediated acute immune surveillance (AIS) via repairable (reusable) DNAs in hrs. Loss of cGAS-mediated AIS decreased the regression of local and abscopal tumours in the context of focal radiation and immune checkpoint blockade. Our results build a direct link between immunosurveillance and DNA mechanical feature.

## Abstract (web summary)

DNA is well-documented to stimulate immune response. Here the authors show that the activation of cyclic GMP-AMP synthase (cGAS) depends on DNA mechanical flexibility, itself determined by DNA-sequence, damage and length.

## Introduction

**Fig. 1:**
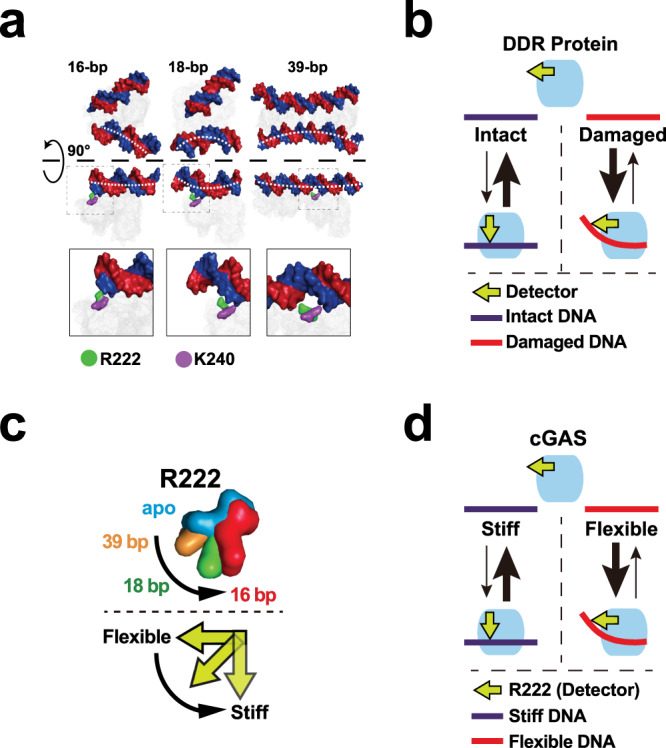
Schematic showing that DNA mechanical flexibility controls cGAS activation. **a** Schematic showing that DNA strands are curved in the 16- (PDB 4K96), 18- (PDB 4LEZ), and 39 bp (PDB 5N6I) DNA–cGAS atomic models. Notably, the conformation of cGAS R222 DNA-binding residue is considerably changed. R222 and K240 bind the same single strand of DNA (blue strand) in 18- and 16 bp atomic models, while R222 and K240 separately bind the distinct single strands of DNA in 39 bp atomic model. DNA and residues are shown by surface representations. Blue and red: DNA; Green: R222; Purple: K240. **b** Schematic showing that DDR protein classically changes the conformation of detector to deform DNA to integrate DFC. It is energetically unfavourable to curve the intact/stiff DNA. Hence, the intact/stiff and damaged/flexible DNAs form unstable and stable protein–DNA complexes, respectively. **c** Upper panel: Superposition analyses of R222 in apo (blue) (PDB 4K8V), 16- (red), 18- (green) and 39 bp (orange) DNA–cGAS atomic models showing that the R222 conformation is changed with the DNA length. The side chain of R222 in apo cGAS atomic model is approximately perpendicular to the side chain of R222 in the 16 and 18 bp DNA–cGAS atomic models. R222 is shown by surface models. Low panel: Schematic showing that the conformation of R222 (yellow arrow) might be changed with DNA-flexibility, which is highly associated with DNA-length. **d** Schematic showing that, like DDR proteins described in (**b**), cGAS might change the conformation of R222 detector to integrate DFC. Consistently, cGAS binds the stiff and flexible DNA to form the unstable and stable cGAS–DNA complexes, respectively.

DNA is highly immunogenic, but the nature of the DNA to activate immune surveillance (IS) remains elusive. As a central DNA-sensor, cyclic GMP-AMP synthase (cGAS) binds DNA and induces the cGAS–DNA condensation to bypass the concentration threshold^1–3^. Then, activated cGAS synthesizes cyclic GMP–AMP (cGAMP) to mount immune responses, including the interferon (Ifn) production^1,4^. cGAS exclusively binds the DNA phosphate backbone^5^. Hence, cGAS was believed to sense DNA in a sequence-independent way^6,7^, necessitating some mechanism for nonself or dangerous DNA recognition. DNA length was first reported to control the cGAS activation^3,8^. Each cGAS monomer directly binds <16 bp DNA (Fig. 1a), and cGAS_2_–DNA_2_ is the mini unite for cGAS activity^7^. Hence, 20 bp DNA should be sufficient to fully activate cGAS, but it poorly activates even mouse cGAS. The cGAS–DNA ladder model provides an elite explanation for the cGAS length-dependent activation^9^. According to the ladder model, the cGAS2–DNA2 assembly is ineffective and unstable, but it triggers the binding of subsequent cGAS to form the cGAS_2n_–DNA_2_ (*n* ≥ 2) ladder. As a result, the longer DNA should more notably activate cGAS. Interestingly, even if the short-length DNA (<30 bp) can only form cGAS_2_–DNA_2_, the longer DNA still more effectively binds and activates cGAS^3^, suggesting that the cGAS activation might depend on some length-associated DNA feature.

A well-known length-associated feature of DNA is the mechanical flexibility to deform^10^. The association between DNA-length and DNA-flexibility to curve (DFC) is so high that the DFC is almost a function of length^11^. In addition to length, sequence also regulates DFC via the base-stacking and -pairing^12,13^. Because the structural integrity is fundamental to the DNA mechanical stiffness, damages are more potent to elevate DFC than length and sequence^12,13^. Consistently, the detection of damage-increased DFC via curving DNA is central for the DNA damage response (DDR) proteins to bind and identify the lesions^14^. DDR proteins classically change the conformation of a detector domain to curve DNA to integrate DFC (Fig. 1b)^14^. For example, base excision repair enzymes curve a lesion nucleotide out of the helix and occupy the void via a single detector residue such as phenylalanine (F) or arginine (R)^14^. It is energetically unfavourable to curve the intact/stiff DNA. Hence, the intact/stiff and damaged/flexible DNAs form unstable and stable complexes with DDR proteins, respectively^14^. As a result, the overall effect of the detector is to decrease the DDR enzyme-binding and -activating by the stiff DNAs, and/or increase the binding and activating by flexible DNA.

Interestingly, crystal structures suggest that cGAS might curve DNA^5^ (Fig. 1a). And cGAS is more effectively activated by the longer DNAs^3,8^, which generally show higher DFC^10,11^. Moreover, cGAS detects the viruses typically ejecting their DNAs into the nuclei^1,15,16^. Although the wrapped nucleosomal DNA might less likely to activate cGAS^9,17,18^, the linker DNA is still thousands of the viral DNA size. Notably, the nucleosomal DNA sequences usually show high DFC near dyads and low DFC near linkers^13,19^. While to be packaged into tiny virions, viral DNA sequences classically show high DFC^20^, suggesting that cGAS might discriminate nonself DNA via DFC.

Additionally, how to fine-tune IS is central to cancer therapies^21,22^. Daily fractions of therapeutic irradiation (IR) is conventionally applied at a low dose to result in repairable DNA damages to spare normal cells^22^. But, the low-dose IR still triggers IS, selectively kills cancer cells and yields outcomes superior to single high-dose IR^22–27^. Low-dose IR mounts acute immune surveillance (AIS) in hr(s), and then depending on the dose, late immune surveillance (LIS) might occur in days^26,28^. And cGAS has been reported to be central to the LIS against micronucleus in days post IR^28,29^. Moreover, AIS usually regresses in hrs, while LIS lasts for days^26^. Despite the short duration, AIS leads to myeloid cell recruitment and alteration of innate/adaptive immune responses, leading to an association between AIS and therapeutic efficacy^23–25^. Unfortunately, although high-dose IR is well-known to trigger AIS via membrane rupture^30–32^, how the low-dose IR mounts AIS is less understood.

In this study, we show that DNA-flexibility controls the cGAS activation. Consistent with the capacity of DNA sequence to regulate DFC, DNA sequence was show to be able to regulate cGAS activation. Our analyses suggested that, among the DNA-binding residues in the cGAS–DNA atomic models, R222 showed the most considerable conformational change, and the conformation of R222 is changed with the DNA length (Fig. 1c). In line with the high association between DFC and DNA-length, further substitution analyses suggested that, like DDR proteins, cGAS might utilise R222 as a DFC detector to change its conformation to integrate DFC (Fig. 1d). Importantly, in the cGAS indiscriminate model, the presence and absence of DNA itself activates and ceases the IS, respectively. While, cGAS surveillance of flexibility suggests that the reversible flexibility regulators (such as modifications, damages and ionic strength) might control the IS. A notable difference between the two models is that the DNA to mount flexsurveillance might be reusable/repairable. Based on this difference, low-dose IR was found to activate cGAS-mediated AIS potentially via repairable DNAs. And the loss of cGAS-mediated AIS decreased the regression of local and abscopal tumours in the context of focal IR and immune checkpoint blockade. Collectively, our results build a link between immunosurveillance and DNA mechanical features (surveillance), which might be targeted for therapeutic intervention.

## Results

### DNA-flexibility regulated by damages controls cGAS activation

**Fig. 2:**
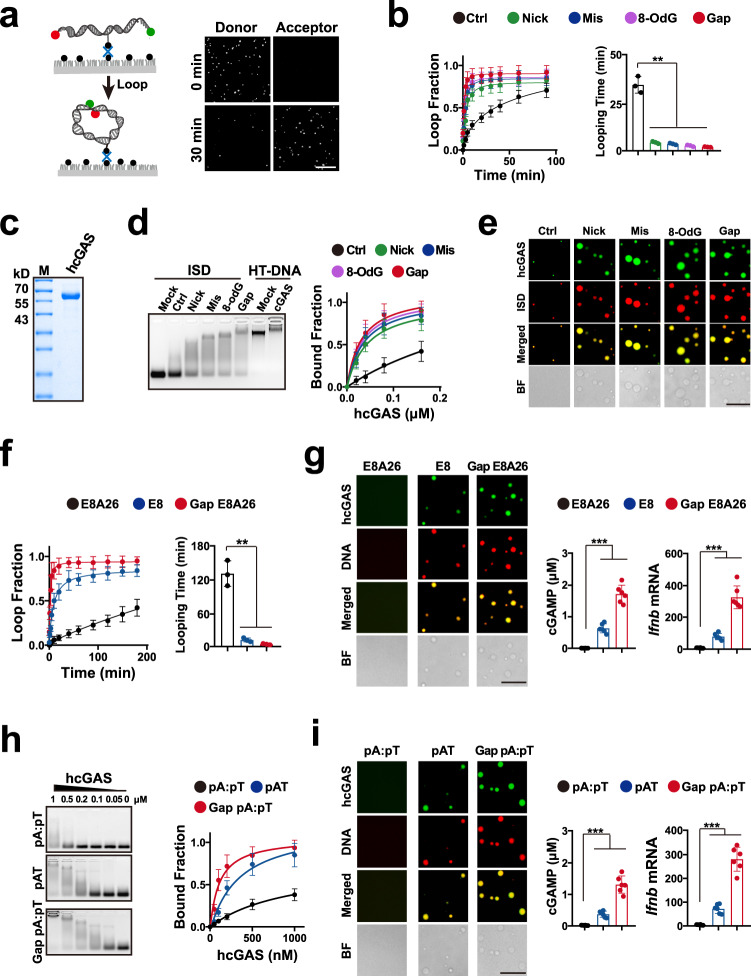
DNA-flexibility controls DNA-potential to bind and activate cGAS. **a** DNA looping assay schematic and fluorescence images of single DNA in donor and acceptor channels after 1 M NaCl treatment for noted time. Scale bar = 10 μm. **b** Loop fraction and time of damaged ISDs. In this study, data are presented as mean values ± standard deviations (SDs), and error bars indicate SDs (Mean ± SD). *n* = 3 independent experiments. **c** Coomassie staining of purified human cGAS (hcGAS) protein. M: Marker; kD: kilodaltons. **d** Left: EMSA of hcGAS binding to noted ISD using HT-DNA as control. Right: EMSA bound fraction of 50 nM ISDs binding to serial dilutions of hcGAS protein. Mean ± SD. *n* = 3 independent experiments. **e** Representative images of hcGAS–DNA condensates after mixing 2 μM hcGAS with 2 μM ISD. Red: Cy3-labelled DNA; Green: Alexa Fluor- 488 (AF-488)-labelled hcGAS; BF: bright field. Scale bar = 20 μm. **f** Looping fraction and times of noted ISDs. Mean ± SD. *n* = 3 independent experiments. **g** Left: Representative images of hcGAS–DNA condensates after mixing 2 μM hcGAS with noted ISD. Red: Cy3-labelled DNA; Green: AF-488-labelled hcGAS; BF: bright field. Scale bar = 20 μm. Middle: cGAMP produced by mixing 100 nM cGAS with 100 nM ISD for 2 h. Right: Ifnb mRNA levels in the MEF cells transfected with 100 ng/mL ISD for 2 h. Mean ± SD. *n* = 6 biologically independent samples. **h** EMSA analyses of 50 nM ISD binding to hcGAS. Mean ± SD. *n* = 3 independent experiments. **i** Left: Representative images of cGAS–DNA condensate formation after mixing hcGAS with noted ISD. Red: Cy3-labelled DNA; Green: AF-488-labelled hcGAS; BF: bright field. Scale bar = 20 μm. Middle: cGAMP produced by mixing hcGAS and ISD. Right: Ifnb mRNA levels in the MEF cells transfected with ISD. Mean ± SD. *n* = 6 biologically independent samples. Unless specifically noted, *P* values were from unpaired two-tailed Student’s *t* test (***p < 0.001, **p < 0.01, *p < 0.05) in this study. Source data are provided as a Source Data file.

We initiated this study to determine which and how DNA feature activates IS. cGAS is central to DNA detection, and structural analyses show a DNA-curving even in cGAS_2_–DNA_2_ dimers (Fig. 1a)^5^, suggesting a role of DNA flexibility to curve (DFC) in the cGAS activation. Hence, we investigated the role of DFC in cGAS activation using multiple sets of DNAs with distinct DFCs. We first assessed the impact of damages on the DFC of same sequence DNAs via looping assay^13,33^, which detects fluorescence resonance energy transfer (FRET) between fluorophores at complementary single-stranded overhangs (Fig. 2a). Because DNA wrapped by nucleosome is unlikely to activate cGAS^9,18,34,35^, a linker sequence (44 bp)^13^ was used to mimic the stiff linker DNAs. Previous studies showed that the energy to form a DNA stem-loop is ~3 kcal/mol^36,37^, while the stacking energetic difference between a normal and mismatched base pair is ~2 kcal/mol^14,38^. Consistently, all four major types of DNA damages (mismatch, nick, 8-hydroxydeoxyguanosine (8-OdG) and gap) reduced the looping time (Fig. 2b), indicating that the increased DFC is a shared feature of these damaged DNAs.

Next, we evaluated the effect of damage-increased DFC on the DNA potential to bind, condense and activate cGAS. Full length human cGAS (hcGAS) protein was purified in vitro (Fig. 2c). Electrophoresis mobility shift assay (EMSA) showed that damages elevated the cGAS–DNA binding (Fig. 2d, Supplementary Fig. 1a). The damage-elevated cGAS–DNA binding was also observed in microscale thermophoresis (MST) assays (Supplementary Fig. 1b), which detect changes in the hydration shell of molecules and measures biomolecule interactions under close-to-native conditions. Based on metabolite- or ligand-induced intrinsic fluorescence quenching of the target protein, metabolite affinity responsive target fluorescence quenching (MARTFQ) showed that damages increased the cGAS–DNA binding (Supplementary Fig. 1c). To assess cGAS–DNA condensation, the Alexa Fluor™ 488 (AF488)-labelled cGAS protein was incubated with the Cy3-labelled oligo immune stimulatory DNAs (ISDs). Fluorescence analyses showed that damages elevated the cGAS–DNA condensation (Fig. 2e).

To examine the cGAS activation in vitro, mass spectrometry (MS) was used to analyze the cGAS-produced cGAMP (Supplementary Fig. 1d). Because both cGAS and ISD concentrations can influence cGAS activation^9^, a fixed cGAS or a fixed ISD concentration was used (Supplementary Fig. 1e). In line with the results of cGAS-binding, damages increased the DNA potential to activate cGAS in vitro (Supplementary Fig. 1f) and in the cellular context (Supplementary Fig. 2a, b). Then, a set of 80 bp ISDs with distinct site, quantity and strand damages^13^ (Supplementary Data 1) were used to further evaluate the potential role of damage-increased DFC in activating cGAS. As shown in Supplementary Fig. 2c, d, all of these damages elevated the DNA potential to activate cGAS. Because a shared feature of these damaged DNAs is their DFC increase, these results suggested cGAS surveillance of DNA-flexibility (flexsurveillance).

### DNA-sequence can regulate cGAS activation via DNA-flexibility

cGAS was previously reported to sense DNA in a sequence-independent way. However, our results suggested cGAS surveillance of DNA-flexibility, which is also controlled by DNA-sequence. To evaluate the role of DNA-flexibility and -sequence, consecutive 26 A bases were embedded into a flexible DNA (E8) to build a reported stiff DNA (E8A26)^33^. These DNAs showed the same length but distinct DFC. The DFC of E8A26 was much lower than that of E8, while gap damage increased the E8A26 DFC (Fig. 2f). Further EMSA (Supplementary Fig. 3a) and MARTFQ (Supplementary Fig. 3b) showed that the E8 and gap-E8A26 DNAs more notably bound cGAS than the stiff E8A26 did. Consistently, the E8 and gap-E8A26 effectively condensed and activated cGAS, while the stiff E8A26 DNA could not (Fig. 2g). Hence, DNA-sequence might control cGAS activation.

To exclude the influences of GC content and looping assay, we assessed the cGAS activation by 44 bp flexible poly(dA:dT) (pAT) and stiff poly(dA):poly(dT) (pA:pT) DNAs, which contain the same GC content. pAT is a repetitive double-stranded DNA sequence of poly(dA-dT):poly(dT-dA) and able to activate cGAS^4^. While, pA:pT was generated by annealing a single-stranded poly(dA) with poly(dT). Because DFC controls the wrapping of nucleosomal DNA^13,19^, it is well-documented that flexible pAT can be effectively wrapped by nucleosome, while stiff pA:pT cannot^39^. In line with the results of E8/E8A26 DNA set, pAT and gap pA:pT DNAs effectively bound, condensed and activated cGAS, while the stiff pA:pT could not effectively activated cGAS (Fig. 2h, i, Supplementary Fig. 3c). Consistently, gap pG:pC and flexible pGC DNAs notably bound and activated cGAS, while the stiff pG:pC could not (Supplementary Fig. 3d–f), suggesting that sequence might control cGAS activation via DFC.

**Fig. 3:**
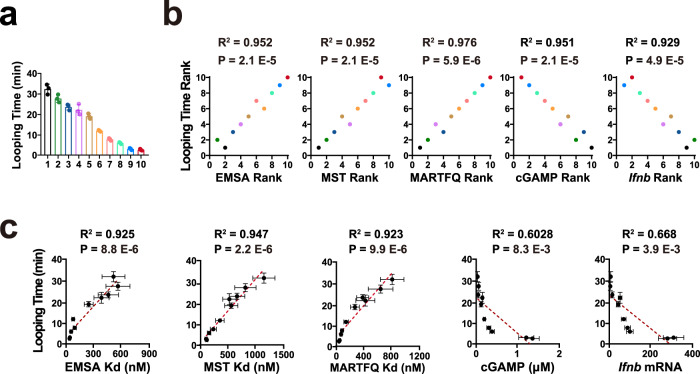
DNA flexibility is associated with DNA potential to bind and activate cGAS. **a** Looping time of seq1–10 ISDs. Mean ± SD. *n* = 3 independent experiments. **b** Spearman analyses of the correlation between the ISD looping time and noted cGAS-binding or -activating potential. *P* and R values were from two-sided spearman analyses. **c** Pearson analyses of the correlation between the DNA looping time and noted cGAS-binding or -activating potential. P and R values were from two-sided Pearson analyses. Red dashed lines are the best fit lines from the linear regression. Mean ± SD. Looping time: *n* = 3 independent experiments; EMSA, MST, MARQTFQ cGAMP and Ifnb mRNA: *n* = 6 biologically independent samples. Source data are provided as a Source Data file.

To further examine the role of DFC and sequence, ten reported 50 bp DNAs (seq1–10) with distinct DFC were used (Fig. 3a)^13^. The potentials of these DNAs to bind and activate cGAS were then assessed (Supplementary Fig. 4a–e). Although DNA sequence has long been known to influence DNA flexibility, prediction of DNA flexibility based on sequence is still a substantial challenge^11,13^. Consistently, the GC content has been well-documented to poorly correlate with DFC (looping time)^11,13,33^, despite of the central impact of GC content on the energy of base pairing. Hence, the GC content was used as a negative control (Supplementary Fig. 4f). As previously described^13,33^, the GC content was not significant (R^2^ = 0.346, *P* = 0.081) correlated with the DFC (Supplementary Fig. 4g). Accordingly, spearman analyses indicated that the GC content was poorly (*P* > 0.05) correlated with the DNA potentials to bind or activate cGAS (Supplementary Fig. 4h). While, spearman analyses showed that DFC was significantly (*P* < 0.0001) associated with the DNA potential to bind and activate cGAS (Fig. 3b), suggesting that sequence might control cGAS activation via flexibility. Consistent with the results from Spearman analyses, Pearson analyses indicated that DFC (looping time) significantly correlated with the potentials of DNA to bind and activate cGAS (Fig. 3c). Interestingly, Pearson analyses showed that cGAS-binding potential more significantly correlated DFC (looping time) than cGAS-activating potential did (Fig. 3c), in line with the threshold effect in cGAS activation^3,9^.

### Substitution analyses identify a role of R222 in the DNA-flexibility detection

**Fig. 4:**
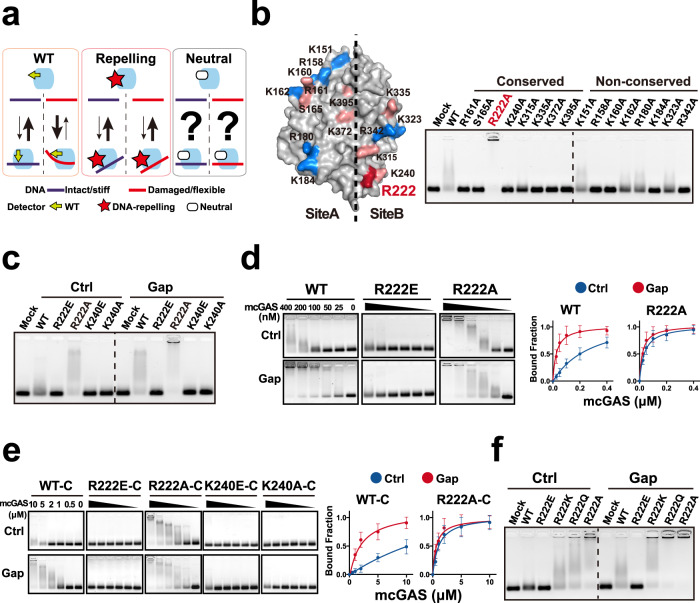
Substitution analyses identified a role of R222 in the DNA-flexibility detection. **a** Schematic showing the potential impacts of detector mutations on the protein–DNA binding. DDR protein classically changes the conformation of detector to deform DNA to integrate DFC. It is energetically unfavourable to curve the intact/stiff DNA. Hence, the intact/stiff and damaged/flexible DNAs form unstable and stable protein–DNA complexes, respectively. And the overall effect of detector might decrease the enzyme-binding and -activating by intact DNA, and/or increase the binding and activating by damaged DNA. The substitutions of these residues to acidic glutamine (E) usually show the DNA-repelling potentials. But, the neutral substitutions might increase but not decrease the enzyme-binding and/or activating by DNAs, providing a strategy to evaluate the role of single residue of cGAS. **b** Left: Surface structure of mcGAS to show the location of DNA-binding residues. The atomic model of 18 bp DNA–cGAS complex (4LEZ) was visualized by PyMOL. The residues conserved and non-conserved between human and mouse are shown in pink and blue, respectively. Red: R222 residues. Right: EMSA analyses of 50 nM 44 bp ISDs binding to 300 nM noted full length neutral mcGAS mutants. Red: R222 residues. **c** EMSA analyses of 50 nM 44 bp ISDs binding to 100 nM noted full length mcGAS proteins. **d** EMSA analyses of 50 nM 44 bp ISDs binding to serial dilutions of mcGAS proteins. Mean ± SD. *n* = 3 independent experiments. **e** EMSA analyses of the 44 bp ISDs binding to serial dilutions of noted C-terminal (catalytic domain) truncated mutant mcGAS proteins. Mean ± SD. *n* = 3 independent experiments. **f** EMSA analyses of 50 nM 44 bp ctrl or gap ISD binding to the noted mcGAS. Source data are provided as a Source Data file.

To investigate the mechanism of cGAS flexsurveillance, we then determined which domain(s) or residue(s) of cGAS detects the DFC. As mentioned above, DDR protein classically has a detector to curve DNA to integrate DFC^14^. Notably, the overall effect of the detector decreases the enzyme-binding and -activating by the intact DNAs, and/or increases the binding and activating by damaged DNA. For example, base excision repair enzymes curve a lesion nucleotide out of the helix, and occupy the void via a residue such as R177 residue of apurinic/apyrimidinic endonuclease 1 (APE1)^14^. The substitutions of these residues to acidic glutamine (E) usually show the DNA-repelling potentials. Importantly, although the detector residue itself might show DNA-binding potential, the neutral substitutions such as APE1 R177A might increase but not decrease the enzyme-binding and/or activating by DNAs^40^. This is consistent with the overall effect of detector to decrease but not increase the enzyme-binding and -activating by intact DNAs. Hence, the neutral substitution was used as a strategy to evaluate the role of single residue of cGAS (Fig. 4a). Interestingly, cGAS is quickly evolved across mammals^41^, but damages elevated the DNA potential to activate both human hcGAS and mouse mcGAS, suggesting that the detector might be conserved. Moreover, cGAS can form the stable cGAS_1_–DNA_1_ via siteA, while the cGAS_2_–DNA_2_ assembly via both siteA and siteB is unstable^5,9^. Because, the detector should lead to unstable DNA–cGAS binding, the siteB residue(s) might show more possibility to be the detector.

All of the DNA-binding residues^5^ were then mutated to neutral alanine (A) to evaluate their role in the mcGAS activation by 44 bp DNAs. The proteins of cGAS residue substitution mutants were expressed and purified in vitro. Coomassie gel analyses of the resultant proteins suggested that the purity of the recombinant proteins was sufficient to the experiments (Supplementary Fig. 5a). Circular dichroism (CD) and dynamic light scattering (DLS) analyses indicated that the integrity of the recombinant proteins might be also sufficient to the further study (Supplementary Fig. 5b, c). EMSA showed that only the substitution of a conserved siteB residue R222 (R222A) increased the cGAS–DNA binding (Fig. 4b), although K240 is pretty close to R222 (the distance between K240 and R222 main-chain N (nitrogen) atoms: ~6.5 Å). Conversely, the DNA-repelling R222E and K240E reduced DNA-binding as previously described^5^ (Fig. 4c). And the DNA-repelling potential of R222E was confirmed by the observations that R222E reduced the ≥ 500 bp DNA binding (Supplementary Fig. 5d). Notably, R222 substitution reduced the mcGAS-binding difference between intact/stiff and gap/flexible DNAs (Fig. 4d), suggesting a contribution of R222 to DFC detection. Consistent with the in vitro results, R222 substitution also reduced the intracellular mcGAS-activating difference between intact/stiff and gap/flexible DNAs (Supplementary Fig. 5e, f). Interestingly, as previously described^5,42^, R222E expressed at low level could not be activated in the cellular context, while R222E expressed at high level could be activated (Supplementary Fig. 5f). This observation might be explained by the previous studies showing that nucleosome binds R222 (human R236) to inhibit cGAS activation, and R222E reduces the cGAS inhibition by nucleosome^18,34,35,42,43^. We speculated that, in the cellular context, R222E might reduce the capacity of nucleosome to inhibit cGAS activation, and the overexpression of R222E might bypass the concentration threshold for R222E to be activated. Furthermore, we also evaluated the role of R222A in the dimerisation of mcGAS. Native gel analyses indicated that R222A showed little impact on the mcGAS dimerisation (Supplementary Fig. 5g). Consistent with the results of full-length cGAS, R222A also reduced the difference between intact/stiff and gap/flexible DNAs to bind the cGAS catalytic domain (C-terminal) truncated mutant (Fig. 4e, Supplementary Fig. 5h), raising a role of R222 in flexibility detection.

Next, R222 was mutated to K (basic but shorter and more flexible than R) and Q (neutral with similar structure as R) to evaluate the impacts of charge and steric effects, respectively (Supplementary Fig. 5i). As shown in Fig. 4f, R222Q and R222A showed comparable potential to bind DNA, indicating a poor steric effect. While, R222Q and R222A more notably increased the intact DNA–cGAS binding than R222K did, suggesting that the charge of R222 contributed to the detection of DFC. Interestingly, R222K showed more potential to bind intact DNA than control (wild type, WT) did, suggesting an impact of the length and conformational stiffness of R residue.

To further evaluate the role of R222 in detecting DFC, multiple sets of DNAs with the same length but different DFC were applied. R222 substitution neutralized the difference between intact/stiff and damaged/flexible DNAs to bind mcGAS (Supplementary Fig. 6a). In line with the results of intact-damaged DNAs, R222 substitution neutralized the binding difference between stiff and flexible DNAs in the context of E8A26/E8 and pA:pT/pAT DNA sets (Supplementary Fig. 6b, c). Consistently, R222 substitutions also reduced the difference between stiff and flexible DNAs to activate the in vitro (Supplementary Fig. 7a–c) and intracellular (Supplementary Fig. 7d–f) cGAS.

Additionally, to determine whether the role of mouse R222 is conserved between human and mouse, the human R236 (corresponding to mouse R222) were mutated to E and A (Supplementary Fig. 8a–c). In line with the results of mouse R222, the human R236 substitutions also reduced the difference between stiff and flexible DNAs to bind and activate hcGAS (Supplementary Fig. 8d–i), indicating a conserved role of mouse R222.

### Human R236 substitution relaxes the DNA-length specificity of cGAS

**Fig. 5:**
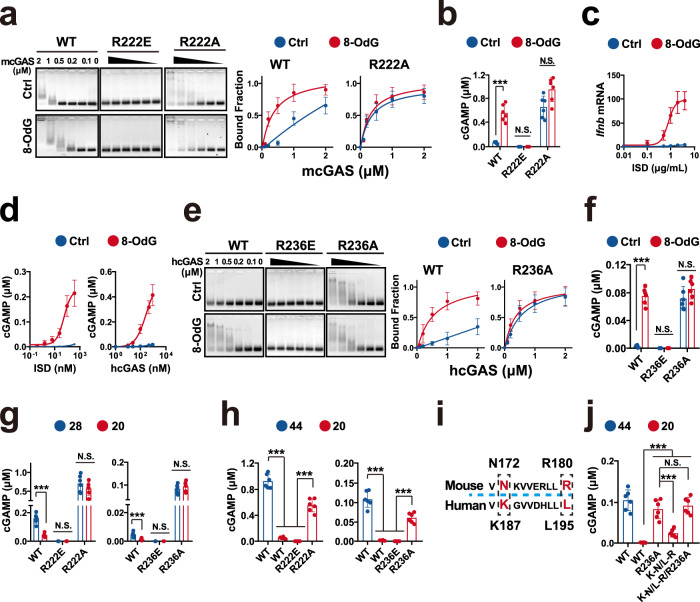
Human R236 substitution relaxes the DNA-length specificity of cGAS. **a** EMSA analyses of 50 nM 20 bp ISDs binding the noted mouse mcGAS proteins. Mean ± SD. *n* = 3 independent experiments. **b** cGAMP produced by mixing 100 nM noted mcGAS and 100 nM 20 bp noted ISD for 2 h. Unless specifically noted, P values were from unpaired two-tailed Student’s *t* test without adjustment for multiple comparisons (***p < 0.001, **p < 0.01, *p < 0.05, N.S. = not statistically significant (*p* > 0.05)) in this study. Mean ± SD. *n* = 6 biologically independent samples. **c** Ifnb mRNA levels in the human THP-1 cells transfected with 100 ng/mL 20 bp noted ISDs for 2 h. Mean ± SD. *n* = 3 biologically independent samples. **d** Left: cGAMP produced by 100 nM human hcGAS in serial dilutions of noted ISD for 2 h. Right: cGAMP produced by serial dilutions of human hcGAS in 100 nM noted ISD for 2 h. Mean ± SD. *n* = 3 biologically independent samples. **e** EMSA analyses of 50 nM 20 bp ISDs binding to the noted human hcGAS proteins. Mean ± SD. *n* = 3 biologically independent samples. **f** cGAMP produced by mixing 100 nM noted human hcGAS and100 nM 20 bp noted ISD for 2 h. Mean ± SD. *n* = 6 biologically independent samples. **g** cGAMP produced by 100 nM noted mouse mcGAS (left) or human hcGAS (right) in the presence of 100 nM 28- or 20 bp ISD for 2 h. Mean ± SD. *n* = 6 biologically independent samples. **h** cGAMP produced by 100 nM noted mouse mcGAS (left) or human hcGAS (right) in the presence of 100 nM 44- or 20 bp ISD for 2 h. Mean ± SD. *n* = 6 biologically independent samples. **i** Sequence alignment to show that the human K185 and L195 residues are non-conserved between human and mouse. **j** cGAMP produced by 100 nM noted human hcGAS in the presence of 100 nM 44- or 20 bp ISD for 2 h. Mean ± SD. *n* = 6 biologically independent samples. Source data are provided as a Source Data file.

Because the DFC is almost a function of DNA-length, we then evaluated the impact of the detector on the DNA-length potential to activate cGAS. To diminish the influences of the cGAS-DNA oligomerization, we investigated the role of the detector in activating cGAS by the different length DNAs, which can only form cGAS_2_–DNA_2_. We first determined whether mouse R222 (human R236) senses the flexibility of 20 bp DNAs. In line with the results of 44 bp DNA, R222 substitutions revoked the difference between stiff and flexible DNAs to bind and activate mcGAS (Fig. 5a, b). Consistent with the results of mouse mcGAS, damage also enhanced the potential of 20 bp DNA to activate the human hcGAS in the cellular context (Fig. 5c) and in vitro (Fig. 5d). The substitution of human R236 reduced the difference between 20 bp stiff and flexible DNA to bind and activate the human hcGAS (Fig. 5e, f). Hence, mouse R222 (human R236) might sense the flexibility of the short DNAs, which can only form cGAS_2_–DNA_2_.

To further evaluate the role of mouse R222 and human R236, we examined the activation of cGAS by the different length DNAs, which can only form cGAS_2_–DNA_2_. Consistent with a previous study^3^, even if the DNA < 30 bp can only form cGAS_2_–DNA_2_, the 28 bp DNA still more effectively activated cGAS than 20 bp DNA (Fig. 5g). While, the R222 (R236) substitutions reduced the difference between short and long DNAs to activate cGAS (Fig. 5g), suggesting a role of R222 (R236) in the length-potential to control cGAS activation.

cGAS activation is controlled by the DNA length^9^. Each cGAS monomer directly binds <16 bp DNA, and cGAS_2_–DNA_2_ is the minimal catalytic unite^9^. Hence, 20 bp DNA should be sufficient to fully activate cGAS, but it poorly activates even mouse cGAS. Conversely, the longer DNA such as 44 bp can more effectively activate cGAS^9^. The cGAS–DNA ladder model provides an elite explanation for the cGAS length-dependent activation^9^. According to ladder model, the cGAS2–DNA2 assembly is ineffective and unstable, but it triggers the binding of subsequent cGAS to form the cGAS2n–DNA2 (n ≥ 2) ladder. As a result, the longer DNA should more notably activate cGAS. Consistently, 20 bp DNA can form cGAS2–DNA2 complex, while 44 bp DNA can form cGAS4–DNA2 complex^9^. And 44 bp DNA had more potential to activate WT cGAS (Fig. 5h). Interestingly, the activation of neutral mutants by 20 bp DNA was comparable with the activation of WT cGAS by 44 bp DNA (Fig. 5h). Because the DNA-length threshold to activate human hcGAS is ~40 bp^3,8,9^, these results suggested that detector neutral substitution might relax the DNA-length specificity of hcGAS. Previous study has showed that human L195 (corresponding to mouse R180) and K187 (mouse N172) at siteA can relax the DNA-length specificity to activate hcGAS^17^. Hence, we used K187N\L195R as a positive control (Supplementary Fig. 9a–c). As shown in Fig. 5i, both R236A and K187N\L195R increased the 20 bp DNA potential to activate cGAS, but not significantly additively. While, R236A more effectively increased the short DNA potential to activate cGAS than K187N\L195R, indicating a role of R236 in length specificity of cGAS.

### Structural analyses suggest that R222 detects DNA-curving to sense flexibility

As mentioned above, the detection of damage-increased DFC is central for DDR proteins to identify the lesions^14^. And DDR proteins usually change the conformation of the detector to curve DNA to integrate DFC^14^. Given that the crystal structure of a DDR protein–DNA complex is usually most energetically favourable, a classical strategy to uncover the detector is to identify the structural difference and then validate the candidate detector via residue substitution^14^. To investigate the mechanism for R222 contribution to DFC detection, computationally structural analyses were performed. Because the DFC is almost a function of DNA length, the different-length DNA–cGAS atomic models were used to evaluate the structure of mcGAS bound by the distinct DFC DNAs. The 16-, 18-, and 39 bp DNA–mcGAS atomic models (PDB 4K96, 4LEY, and 5N6I, respectively) were applied to computationally evaluate the conformational changes with the DFC^5,9,44^. While an apo structure (PDB 4K8V) was taken as the apo structure of cGAS without DNA-binding^44^.

**Fig. 6:**
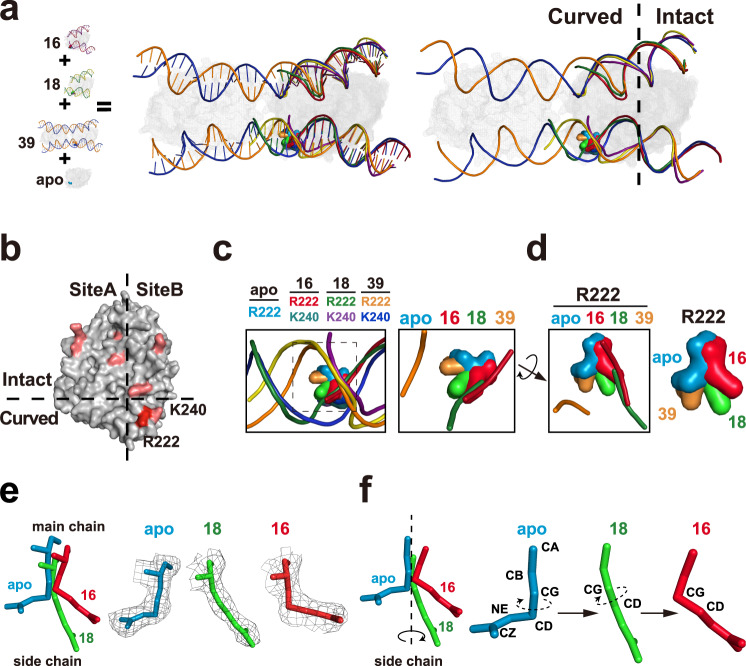
Structural analyses suggest that R222 might detect DNA-curving to sense flexibility. **a** Left: Superposition of apo-, 16-, 18- and 39 bp DNA–cGAS atomic models visualized by PyMOL. The K240-binding single-strand (Kbss) of DNA and the strand complementary to K240-binding single-strand (sc–Kbss) are shown by ribbon representations. The cGAS proteins are shown by mesh representations. Unless specifically noted, R222 residue binds to the corresponding DNA single-strand marked by the same color. For instance, R222 (red) residue binds sc–Kbss (red) in 16 bp atomic model. Right: The main chains of DNAs are shown by ribbon representations, while the side chains of DNAs are hided. Apo: R222: light blue. 16 (16 bp complex): R222: red; K240: dark green; Kbss: red; sc–Kbss: purple. 18: R222: green; K240: purple; Kbss: green; sc–Kbss: yellow. 39: R222: orange; K240: blue; Kbss: blue; sc–Kbss: orange. **b** Surface structure showing the location of conserved DNA-binding residues. **c** Enlarged superposition of the noted atomic models in (**a**). The main chains of DNA are shown by ribbon representations, while the side chains are hided. Unless specifically noted, R222 residue binds to the corresponding single-strand marked by the similar color. For instance, R222 (red) residue binds sc–Kbss (red) in 16 bp atomic model, while R222 (orange) binds sc–Kbss (orange) in 39 bp atomic model. **d** Rotated superposition described in (**c**) to show the colocalization between R222 (surface representations) and DNAs (ribbon representations). **e** The electronic densities of R222 in apo, 16, and 18 atomic models. Due to the resolution limitation, the electronic density of R222 in 39 atomic model was not used for the side chain analyses. Blue: R222 in apo complex; Green; R222 in 18 bp complex; Red; R222 in 16 bp complex. **f** Side chain analyses suggested a potential clockwise rotation of R222 in response to the different-length DNA-binding. Evidenced by the R222 electronic densities described in (**e**), the CG-CD bond is perpendicular to guanidyl group in the apo atomic model, while parallel to guanidyl in 16 atomic model. Blue: R222 in apo complex; Green; R222 in 18 bp complex; Red; R222 in 16 bp complex.

Computationally superposition analyses were performed to evaluate the conformational changes. DNA superposition showed that the 16-, 18-, and 39 bp DNAs were almost overlapped in the DNA intact domain, but they showed notable differences in the curved domain (Fig. 6a, Supplementary Fig. 10a). Because damages elevated the DNA potential to activate both human hcGAS and mouse mcGAS, the residue to detect DFC should be likely conserved. Conserved residue superposition indicated that, in the conserved DNA-binding residues^5^, only R222 and K240 siteB residues were localized in the DNA curved zone (Fig. 6b, Supplementary Fig. 10b). Apo atomic model was then used to evaluate the apo structure of cGAS. As shown in Supplementary Fig. 10c, d, both R222 and K240 show conformational differences between apo and 16 bp atomic models, but only R222 displays a notable difference between apo and 18 bp atomic models. Notably, R with a guanidyl group usually shows less conformational flexibility than K. These results suggested that the R222 conformation might be changed with DNA binding.

The correlation between the DNA-binding and R222 conformational change was then examined. Superposition of DNA and residues showed that the R222 conformations were changed with different-length DNA-binding (Fig. 6c, d, Supplementary Fig. 10e). R222 side chain might show a rotation from the apo (Fig. 6e, f). As a result, the CG-CD bond is perpendicular to guanidyl group in the apo, while parallel to guanidyl in 16 bp atomic model (Fig. 6e, f). Hyperchem analyses indicated that these CG-CD bond rotations might show >5 kcal/mol energetic changes. Hence, the energy from cGAS R222 conformational changes might be comparable with the DNA-curving energy^36,37^, suggesting a possibility to change R222 conformation to curve DNA.

**Fig. 7:**
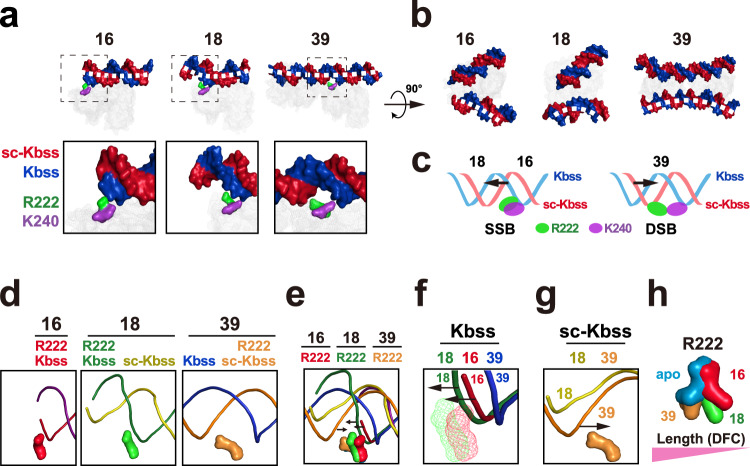
R222 might curve DNA to sense flexibility. **a** Surface structures showing that R222 (green) and K240 (purple) might bind the same DNA single strand (Kbss) in 16 and 18 atomic models, while R222 and K240 might separately bind the distinct single strands (Kbss and sc–Kbss) in 39 atomic model. The DNA Kbss and sc–Kbss are shown by the red and blue surface models, respectively. The cGAS proteins are shown by mesh representations. Blue and Red: DNA; Green: R222; Purple: K240. **b** Surface structures showing that the DNAs are curved to different direction in 16, 18 and 39 atomic models. The DNA Kbss and sc–Kbss are shown by the red and blue surface models, respectively. The cGAS proteins are shown by mesh representations. Blue and Red: DNA. **c** Schematic showing that the distinct-strand-binding (DSB) and same-strand-binding (SSB) models might curve the sc-Kbss (red) and Kbss (blue) single strands, respectively. Blue and pink: DNA; Green: R222; Purple: K240. **d**, **e** Superposition showing that the DNA conformational changes might be correlated with the R222-binding. The R222 residue binds to the corresponding single-strand marked by the same color. For instance, R222 (red) binds sc–Kbss (red) in 16 bp atomic model, while R222 (orange) binds sc–Kbss (orange) in 39 bp atomic model. **f**, **g** Enlarged superposition in (**e**) to show the conformational changes of Kbss (**f**) and sc-Kbss (**g**) DNA single strands might be correlated with the R222-binding. The R222 residue binds to the corresponding single-strand marked by the same color. The R222 residues in (**f**) are shown by mesh representations. **h** Schematic showing that the R222 conformational changes might be correlated with the DNA-length, which is highly associated with DFC. Notably, the side chain of R222 in apo cGAS atomic model is approximately perpendicular to the side chain of R222 in the 16 and 18 bp DNA–cGAS atomic models. R222 is shown by surface models. Blue: R222 in apo complex; Yellow: R222 in 39 bp complex; Green: R222 in 18 bp complex; Red: R222 in 16 bp complex.

Next, we evaluated the correlation between DNA conformational change and R222/K240-binding. The DNA overhang at R222/K240 in 18 bp atomic model is longer than that in 16 bp atomic model (Fig. 7a), suggesting that the DFC in 18 bp atomic model might be higher than that in 16 bp atomic model. Consistently, 18 bp DNA is more notably curved than 16 bp DNA (Fig. 7b, c). Moreover, R222 and K240 separately bind the distinct single strands of DNA (distinct-strand-binding (DSB)) in 39 bp atomic model, while R222 and K240 bind the same single strand of DNA (same-strand-binding (SSB)) in 18- and 16 bp atomic models (Fig. 7a). Although the DNA overhang at R222/K240 in the 18 bp atomic model shows the enough length to be bound, R222 and K240 still display SSB but not DSB (Fig. 7a), suggesting that DSB might require more DFC and/or DNA-length. Notably, the SSB and DSB potentially curve the K240-binding single-strand (Kbss) and strand complementary to K240-binding single-strand (sc–Kbss), respectively (Fig. 7c). And the Kbss of 18 bp DNA (green) shows more conformational change than the 16 bp Kbss (red) (Fig. 7d–f), consistent with the observations that the long DNA usually displays higher DFC than the short DNA. Interestingly, the Kbss DNAs are arranged in an 18–16–39 but not 39–18–16 manner (Fig. 7f), while the sc–Kbss DNAs are arranged in an 18–39 manner (Fig. 7g), suggesting a correlation between DNA conformational change and R222-binding.

Interestingly, superimposition analyses of three cGAS–DNA structures showed that the DNA-binding residues and DNA strands were almost overlapped, suggesting that the free energy (ΔG) of DNA–cGAS binding in three structures should be similar. Notably, it is energy-consuming to curve DNA, which might be a critical reason for the difference of the ΔG of DNA–cGAS binding in 16-, 18- and 39 bp DNA–cGAS complexes. Because DFC is central to the energy to curve DNA^11^, the energy to curve the flexible DNA should be lower than that to curve the stiff DNA. This principle provides a possibility to potentially compare the DNA-flexibility via assessing the free energy (ΔG).

We then performed molecular dynamics (MD) simulations and assessed the free energy (ΔG) of the cGAS–DNA binding in 16-, 18- and 39 bp DNA–cGAS atomic models via molecular mechanics Poisson-Boltzmann surface area (MMPBSA) analyses. Free energy (ΔG = H–TS) represents the ability for a system to do work. For DNA curving, lower ΔG represents more flexibility to be curved. As shown in Supplementary Fig. 11a, the overall ΔG of cGAS–DNA binding in 39 bp DNA–cGAS atomic model < the overall ΔG in 18 bp atomic model < the overall ΔG in 16 bp atomic model, consistent with the observations that longer DNA can more effectively bind and activate cGAS^9^. Due to the pivotal role of DNA flexibility in the energy to curve DNA, these results suggested that the flexibility of 39 bp DNA > the flexibility of 18 bp DNA > the flexibility of 16 bp DNA. Moreover, we also analysed the ΔG of each DNA base in the DNA of 16 bp, 18 bp and 39 bp DNA–cGAS structures. As shown in Supplementary Fig. 11b–d, the ΔGs of most residue-bound bases in 16 bp, 18 bp and 39 bp DNA–cGAS structures were relatively consistent. Notably, the ΔG of R222-bound base(s) in 16 bp and 18 bp structures is >0, but the ΔG of R222-bound base is considerably decreased in 39 bp structure (Supplementary Fig. 11d), in line with the potential role of R222 in detecting DNA-flexibility.

Additionally, the unique structure of 39 bp DNA–cGAS (cGAS_4_–DNA_2_) complex^9^ might further support a role of R222 in the DFC detection. Given that DNA-length considerably elevates the DFC, it should be more energetically favourable to curve a long overhang DNA at R222 than a short overhang DNA (Supplementary Fig. 11e). Hence, cGAS–DNA complex with a longer overhang DNA at R222 might be more stable (Supplementary Fig. 11e). If R222 end of cGAS is taken as head and the other end is taken as tail, the two cGAS dimers in 39 bp DNA–cGAS complex are arranged in a head-to-head orientation to form the cGAS_4_–DNA_2_ structure^9^ (Supplementary Fig. 11f). If the stable cGAS–DNA binding occurs anywhere along the DNA, the two cGAS dimers arranged in head-to-tail or tail-to-tail orientation should be detected. However, only head-to-head orientation was observed in the cGAS_4_–DNA_2_ complex^9^. Notably, two cGAS dimers arranged in head-to-head orientation can result in the maximum length of overhang DNA at R222 of the four cGAS monomers (Supplementary Fig. 11f), suggesting that cGAS–DNA complex with a longer overhang DNA at R222 might be more energetically favourable.

Collectively, these analyses showed that R222 is localized in the curved zone, and displays conformational changes with DNA-binding. The DNA-curving extent is correlated with the R222 conformations, and the conformational energy of R222 is comparable with the DNA-curving energy. Notably, R222 conformational changes are negatively correlated with the DNA-length, which is highly associated with DFC (Fig. 7h). Hence, these results suggested that cGAS might apply R222 to curve DNA to sense DFC.

### Flexsurveillance model suggests that low dose IR might activate cGAS-mediated AIS via repairable DNA

Flexsurveillance model was then applied to deduce which IS(s) with unknown mechanism might be mediated by cGAS. In the indiscriminate model, the presence and absence of DNA itself activates and ceases the IS, respectively. While, flexsurveillance suggests that the presence and removal of reversible flexibility regulator (such as modifications (8-OdG), damages, and ionic strength) can also activates and ceases the IS, respectively. Hence, a notable difference between these two models is that the DNA to activate flexsurveillance might be reusable or repairable. Interestingly, the low dose IR results in relatively repairable DNA damages, triggers AIS, and is well tolerated by normal cells^22–27^. Consistently, ultraviolet (UV) IR in sunlight is well-known to mount AIS, but low dose IR at minimal erythema dose (MED) or sub-MED might even potentially benefit health^45,46^, suggesting that the low dose IR-promoted DNA damages might be repairable. However, little is known about how the low-dose IR mounts AIS.

**Fig. 8:**
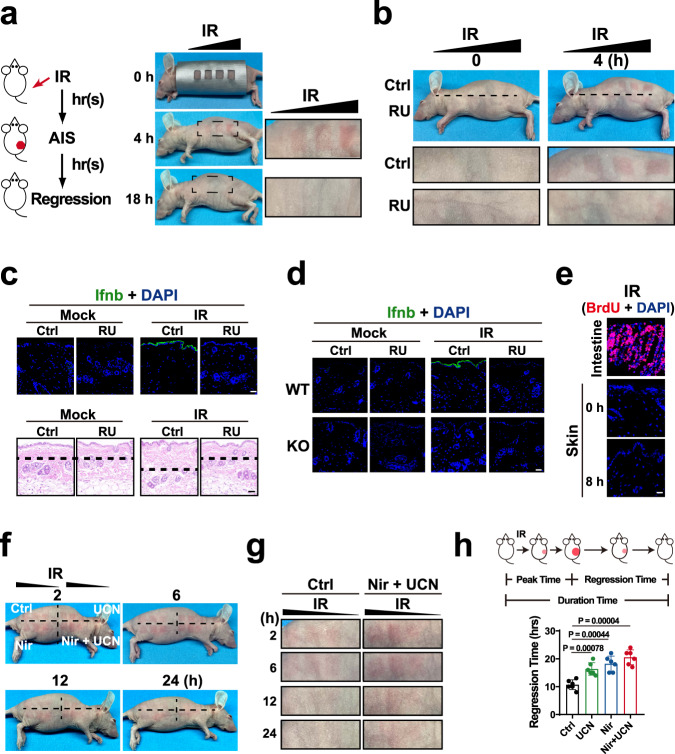
Flexsurveillance model suggests that low dose IR might activate cGAS-mediated AIS via repairable DNA. **a** Left: Schematic showing the time schedule of acute immune surveillance (AIS) and late immune surveillance (LIS). Right: Representative images of the nude mice at noted times after irradiation (IR) (ranging 150–600 mJ/cm^2^). **b** Representative erythema images of the mice pretreated with 150 μg/cm^2^ RU.521 for 1 h at noted times after IR (ranging 150–600 mJ/cm^2^). *n* = 6. **c** Skin immunofluorescence (IF) (upper) and hematoxylin-eosin (HE) (lower) staining images of the RU.521-pretreated nude mice at 4 h after 300 mJ/cm^2^ IR. Scale bar = 100 μm. Upper: Blue: Dapi; Green: Ifnb (anti-Ifnb antibody). Lower: Black dashed lines are used to evaluate the swell. **d** Skin IF images of the cGAS wild type (WT) and knock out (KO) mice at 4 h after 400 mJ/cm^2^ IR. Blue: Dapi; Green: Ifnb (anti-Ifnb antibody). Scale bar = 100 μm. **e** IF images of the nude mice treated with intraperitoneal injection of 100 mg/kg BrdU and IR. Intestinal cells that usually undergo rapid proliferation were used as a positive control for the BrdU staining. Skin: 0 and 8 h after the treatment. Intestine: 5 D after the treatment. Blue: Dapi; Red: BrdU. Scale bar = 100 μm. **f** Representative images of 5 μg/cm^2^ UCN-01 and/or niraparib-pretreated mice at the noted times after IR (ranging 150–600 mJ/cm^2^). **g** Enlarged erythema images of the mice in (**f**). **h** Regression times of erythema in the mice treated with 300 mJ/cm^2^ IR. *P* values were calculated by unpaired two-tailed Student’s t-test without adjustment for multiple comparisons. Mean ± SD. *n* = 6 mice. Source data are provided as a Source Data file.

We then determined whether cGAS mediates the AIS mounted by low-dose IR. To mimic the dose of conventional radiotherapy, only ≤ 4-fold of MED of IR were used in this study. To diminish the spatial influence, UV-B IR showing a low penetration potential was applied to mount AIS. Erythema (Fig. 8a) and Ifnb production (Supplementary Fig. 12a) analyses indicated that AIS could be effectively mounted by IR. And immunofluorescent (IF) analyses suggested that Ifnb was produced mainly by keratinocytes (the outermost layer of the skin) (Supplementary Fig. 12a). cGAS inhibitor (RU.521) was used to evaluate the role of mcGAS. To assess the potential impact of RU.521 on DNA damages, we examined the IR-induced cyclobutane pyrimidine dimer (CPD) levels in the skins of mice treated with IR and/or RU.521. Dot blot analyses of CPD demonstrated that RU.521 showed little effect on blocking DNA damages (Supplementary Fig. 12b). But, RU.521 abrogated the IR-mounted erythema, Ifnb production, and swell (Fig. 8b, c). Consistently, knockout (KO) of mcGAS also blocked IR-mounted AIS (Fig. 8d, Supplementary Fig. 12c). Further MS analyses showed that IR induced mcGAS to product cGAMP, while mcGAS inhibitor or KO revoked the IR-induced cGAMP production (Supplementary Fig. 12d, e), indicating that cGAS mediates low dose IR-mounted AIS.

We then evaluate the potential impact of micronuclei on the AIS. Previous study demonstrated that it takes IR > 3 d to induce a micronucleus-mounted surveillance (LIS) in vitro^28^, suggesting little impact of micronuclei on the AIS in vivo. To further evaluate the role of micronuclei, 5-bromo-2´-deoxyuridine (BrdU) was injected into the mice to assess the cell cycle of skin cells. Because that cGAS surveillance of micronuclei is mitosis-dependent^28,29,47^ and most skin cells are at G1 or G0 stage, the cells must go through S and subsequent M phases to generate micronuclei to activate cGAS. Since BrdU can be incorporated into the DNA during S phase, the BrdU-staining positive cells should be at S phase or have passed S phase. Intestine cells that usually undergo rapid proliferation were used as a positive control of the BrdU-staining. As shown in Fig. 8e, a considerable number of intestine cells were BrdU-staining positive, indicating that BrdU was effectively stained. But BrdU was undetectable in the skin even at 8 h after IR, suggesting that theses skin cells did not go through S phase and unlikely generated micronuclei to activate cGAS.

The above results showed that AIS was mounted by low-dose IR in hr(s) and last only hrs. This temporal kinetics suggested that cGAS-mediated AIS might be activated by elevated DNA-flexibility, and ceased by DNA repair. Hence, the flexibility changes of the damaged but repairable DNAs might be responsible for activating and then ceasing AIS. We then applied two inhibitors to hamper DNA repair to evaluate the roles of damaged but repairable DNA. Two DDR enzymes, poly (ADP-ribose) polymerases (PARPs) and checkpoint kinase 1, were inhibited by niraparib and UCN-01, respectively. These inhibitors protracted the erythema regression (Fig. 8f–h) and duration times (Supplementary Fig. 12f), and increased the cGAMP (Supplementary Fig. 12g) and Ifnb (Supplementary Fig. 12h, i) production. After two day, the erythema regression and duration times were also protracted by DDR inhibitors in the second round treatment (Supplementary Fig. 12j, k), suggesting that low-dose IR might mount cGAS-mediated AIS via repairable DNA.

### Damage elevates the potential of nucleosomal DNA to bind and activate cGAS

cGAS is recently shown to localize mostly in the nucleus^48,49^, and inhibited by nucleosome^18,34,35,42,43^. But cGAS effectively detects the viruses even in the nuclei^7^, suggesting that the inhibition of cGAS by nucleosome might be vulnerable. Our results indicated that low dose IR might mount cGAS-mediated AIS via repairable DNA. Interestingly, nucleosomal DNA is the most abundant DNA in mammalian cells, and nucleosomal DNA should more frequently be damaged, raising a possibility for nucleosomal DNA to act as a type of damaged but repairable DNA to activate cGAS. This was further emphasized by the observation that full-length cGAS (cGAS FL) more notably bound DNA than cGAS C-terminal (cGAS-C) and N-terminal (cGAS-N) truncated proteins did (Supplementary Fig. 13a)^1^. Consistently, inhibition of full-length cGAS by nucleosome is relatively vulnerable, although cGAS-C is remarkably inhibited by nucleosome^18^.

**Fig. 9:**
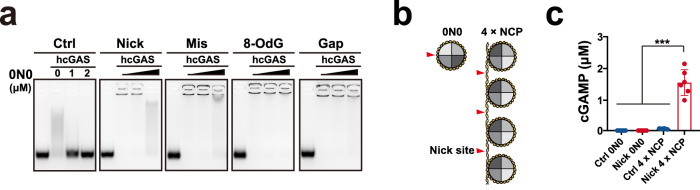
Damages elevate the potential of nucleosomal DNA to bind and activate cGAS. **a** EMSA assays of 50 nM 44 bp Cy3-labelled noted ISD binding to 500 nM cGAS in the presence of serial dilutions of 0N0 (nucleosome-DNA complex with nucleosomal DNA but without linker DNA). Fifty nanometers of 44 bp Cy3-labelled noted ISD and 500 nM human cGAS were mixed with serial dilutions of nucleosome (0N0) that was not fluorescently labelled. After incubation at 37 °C for 30 min, the mixtures were resolved on agarose gel. **b** Schematic showing the nick sites in 0N0 (nucleosome with nucleosomal DNA but without linker DNA) and 4 × NCP (4 nucleosome core particles (NCPs) positioned by Widom-601 sequences spaced by 44 bp linkers). **c** cGAMP produced by 100 nM cGAS in 100 nM noted nucleosomal DNAs for 2 h. *P* values were calculated by unpaired two-tailed Student’s t-test without adjustment for multiple comparisons (*p < 0.05, **p < 0.01 and ***p < 0.001). Mean ± SD. *n* = 6 biologically independent samples. Source data are provided as a Source Data file.

We then evaluated the impact of damages on overcoming the full-length cGAS inhibition by nucleosome. 0N0 structure (nucleosome-DNA complex with nucleosomal DNA but without linker DNA) (Supplementary Fig. 13b) is much more stable than nucleosome (octamer). Hence, 0N0 was alternatively applied to assess the potential of nucleosome. As previously described^18^, EMSA was used to evaluate the competition between nucleosome (0N0) and ISD DNA in binding cGAS. cGAS and fluorescently labelled ISD at fixed concentrations were mixed with serial dilutions of nucleosome (0N0) that was not fluorescently labelled. After incubation, the mixtures were resolved on gel. As shown Fig. 9a, nucleosome (0N0) more effectively disrupted the Ctrl-ISD–cGAS-binding than damaged-ISD–cGAS-binding. Then, cGAS and fluorescently labelled nucleosome (0N0) at fixed concentrations were mixed with serial dilutions of ISD that was not fluorescently labelled. After incubation, the mixtures were resolved on gel. As shown Supplementary Fig. 13c, damaged-ISD more effectively inhibited the cGAS–nucleosome (0N0) binding than Ctrl-ISD did. In the presence of 0N0, damaged-ISDs more effectively activated cGAS than Ctrl-ISD (Supplementary Fig. 13d). In addition, 4 × nucleosome core particles spaced by 44 bp linkers (4 × NCP)^18^ with Nt.BspQI cleaving sites were applied to evaluate the impact of nick damage on the ISP of nucleosomal DNA (Fig. 9b). MS analyses of cGAS-produced cGAMP showed that damaged nucleosomal DNA effectively activated cGAS, while the intact control DNA could not (Fig. 9c), suggesting that low dose IR might mount AIS via repairable nucleosomal DNA.

### Loss of cGAS-mediated daily AIS impairs the radiotherapy efficiency

**Fig. 10:**
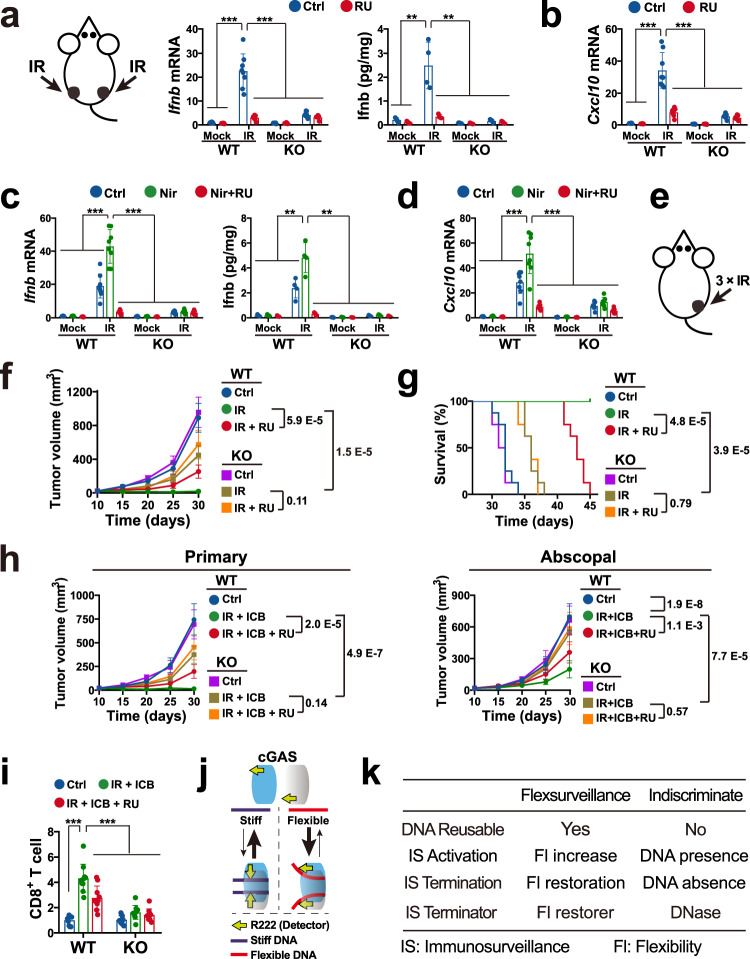
Loss of cGAS-mediated daily AIS impairs the radiotherapy efficiency. **a** Ifnb mRNA (left) and protein (right) levels in the tumours at 4 h after single focal 8 Gy IR. Mean ± SD. MRNA: *n* = 8 tumours from 4 mice. Protein: *n* = 4 tumours from 4 mice. **b** Cxcl10 mRNA levels in the tumours in (**a**). Mean ± SD. *n* = 8 tumours from 4 mice. **c** Ifnb mRNA (left) and protein (right) levels in the inhibitor-pretreated mice at 4 h after single IR. Before IR, mice were injected with 5 mg/kg RU.521 for 3 h or treated by oral gavage with 4 mg/kg of niraparib for 3 h. Mean ± SD. MRNA: *n* = 8 tumours from 4 mice. Protein: *n* = 4 tumours from 4 mice. **d** Cxcl10 mRNA levels in the tumours in (**c**). Mean ± SD. *n* = 4 tumours from 4 mice. **e** Schematic showing right flank implanted tumour treated by three focal fractions of 8 Gy IR on D10, D11 and D12. **f**, **g** Tumour volumes (**f**) and survival percentages (**g**) in the RU.521-pretreated mice described in (**e**). P values in (**f**) were from unpaired two-sided Student’s t-test without adjustment for multiple comparisons. *P* values in (**g**) were from log-rank test. Mean ± SD. *n* = 8 mice. **h** Primary (right flank) and abscopal (left flank) tumour volumes in the mice treated with RU.521 prior to IR. Implanted tumours were treated by three focal IR fractions on the primary tumour, followed by CTLA-4 mAb treatment. Mean ± SD. *n* = 10 mice. **i**, CD8 + T cells in the abscopal tumours described in (**h**). Mean ± SD. *n* = 10 mice (tumours). **j** Schematic showing IS of DNA-flexibility. cGAS might apply R222 to detect the DNA-flexibility via DNA-curving. **k** The differences between flexsurveillance and DNA indiscriminate models. *P* values in (**g**) were from log-rank test, while all other *P* values were from unpaired two-tailed Student’s *t* test without adjustment for multiple comparisons (***p < 0.001, **p < 0.01, *p < 0.05). Source data are provided as a Source Data file.

Due to the role of IS in radiotherapy, we then evaluated the role of cGAS-mediated AIS in the daily fractions of radiotherapy using implanted tumours. The previous study has shown that it takes IR > 3 d to induce a micronucleus-mounted LIS in vitro^28^. To diminish the influence of micronuclei, all of the cGAS inhibitor treatments were performed within 2.5 d after the first fraction of IR. MC38 cells were subcutaneously (s.c.) injected into immunocompetent C57BL/6 mice. As 2 Gy is the conventional low therapeutic dose to trigger erythema^22^, 8 Gy (4-fold of MED) IR was performed as previously described^27^. Single IR treatment activated AIS and led to Ifnb and Cxcl10 productions in 2 h, while loss of cGAS-mediated AIS via cGAS KO or inhibitor-pretreatment blocked the Ifnb and Cxcl10 productions (Fig. 10a, b, Supplementary Fig. 14a). Furthermore, niraparib treatment prior to IR increased the Ifnb and Cxcl10 productions, while cGAS KO or inhibitor-pretreatment abrogated the niraparib-increased productions (Fig. 10c, d, Supplementary Fig. 14b). After a day, similar phenomena were also observed in the second round IR and/or niraparib treatment (Supplementary Fig. 14c), suggesting that IR might mount cGAS-mediated AIS via repairable DNA.

Next, we investigated the role of cGAS-mediated AIS in the local and abscopal anti-cancer efficiencies of radiotherapy. To assess the local effect, MC38 cells were injected into the right flank to generate primary tumours. On day 10 (D10), the primary tumours were irradiated with three focal fractions of 8 Gy. As previously described^50^, deletion of cGAS/Sting pathway (KO) impaired the local anti-cancer effect of IR (Fig. 10e–g). Notably, loss of cGAS-mediated AIS via inhibitor pretreatment also decreased the regression of local tumours, suggesting a role of cGAS-mediated AIS in the local effect (Fig. 10e–g). To evaluate the abscopal effect, the cells were implanted to generate primary (local) tumours in the right flank and slightly asynchronous secondary (abscopal) lesions in the left flank. As previously described^27^, three focal fractions of IR without immune checkpoint blockage (ICB) had no detectable abscopal activity (Supplementary Fig. 14d–g). While, the IR and ICB combination showed an abscopal effect^27^, and cGAS KO reduced the abscopal effect (Fig. 10h, i, Supplementary Fig. 14h). Notably, loss of cGAS-mediated AIS also impaired the regression of the abscopal tumour (Fig. 10h). Therefore, the above results indicated a contribution of cGAS-mediated AIS to both local and abscopal anti-tumour effects.

## Discussion

DNA is well-known to trigger immune response^7^, but which and how DNA feature(s) mount IS remains elusive. Here, we show that DNA flexibility controls the IS via cGAS. These results build a direct link between immunosurveillance and DNA mechanical feature (flexsurveillance) (Fig. 10j), which might be targeted for therapeutic interventions.

Because cytosolic DNA sensors exclusively bind the DNA phosphate backbone but not the bases^5,9,44^, they were previously supposed to sense DNA in a sequence-independent way. Interestingly, the flexsurveillance can be regulated by DNA-damage, -length and –sequence, consistent with their roles in DNA-flexibility^10,11^. Consistently, our study shows that the DNA-sequence can control cGAS activation at least partially via DNA-flexibility.

cGAS flexsurveillance might suggest potential strategies for the therapeutic intervention. Flexsurveillance shows several difference with the DNA indiscriminate model (Fig. 10k)^6^. In the indiscriminate model, the presence and absence of DNA itself activates and ceases the IS, respectively. The indiscriminate model contributed to the understanding of IS activated by the DNAs, including micronucleus, mitochondrial and viral DNAs^7,15,28,51,52^. While, the flexsurveillance suggests that the IS can be controlled by the relatively reversible DNA-flexibility regulator including ionic strength, DNA-modifications (such as 8-OdG and m1dA) and even DNA-damages. A difference between these two models is the potential reusable/repairable DNA to activate flexsurveillance after an on-off IS cycle. As a result, flexsurveillance might enable normal cells to tolerate the multiple on-off cycles of cGAS-mediated IS, suggesting potential strategies for the intervention. This is supported at least partially by our results that low dose IR activates cGAS-mediated AIS, which usually undergoes multi on-off cycles in the context of consecutive daily radiotherapies^22^. And the loss of cGAS-mediated AIS decreased the regression of local and abscopal tumours.

Our results show an immune mechanism for direct DNA damaged–undamaged discrimination. Although DNA damages has long been known to mount immune response^53^, the immune mechanism for damaged DNA discrimination is less elucidated. We show that cGAS detects damaged DNA via flexsurveillance. Although cGAS is one of the most quickly evolved genes in the vertebrates^41^, R222 is almost conserved across the vertebrates (Supplementary Fig. 14i), suggesting a conserved role of flexsurveillance. Moreover, even bacteria apply the elevated DFC to recognise the lesions^11,54^, indicating an ancient role of DFC in detecting the lesions. While, only vertebrates are evolved to acquire cGAS–IFN system^55^, suggesting that a young sensor is quickly evolved to acquire an ancient mechanism for damaged DNA recognition.

Self–nonself recognition is pivotal to IS, thereby cGAS effectively detects the viruses even in the nuclei^1,15,16^. However, little is known about the mechanism for cGAS to recognize nonself-viral DNAs in the nuclei. Interestingly, the nucleosomal DNA sequences show high DFC near dyads and low DFC near linkers, and the wrapping of nucleosomes at the both ends of linkers might further reduce the linker DFC^13,19^. Conversely, viral DNA usually shows high DFC^20^. Our results suggest that flexsurveillance might contribute to nonself DNA discrimination. But more work is still needed to determine the role of flexsurveillance in detecting virus.

This study also reveals an alternative mechanism for the DNA length-dependent activation of IS. Due to the association between DNA-length and DFC^10^, our results suggest that DNA-length might control cGAS activation via DFC. Moreover, the R236 (corresponding to mouse R222) substitution more effectively relaxes the DNA-length specificity to activate human hcGAS than reported K187\L195 substitutions^17^. Interestingly, R236 and K187\L195 could not remarkably additively relax the DNA-length specificity, raising a potential overlapped mechanism for these substitutions. Unlike the conserved human R236, the residues near K187\L195 at siteA are under strong selection even in the primates^41^, suggesting that flexsurveillance might also be fine-tuned in a species-specific way.

However, it should be noticed that there are several limitations of this study. First, DDR proteins usually have a detector domain to change its conformation to curve DNA to integrate DNA-flexibility^14^. A classical strategy to uncover the detector of DDR proteins is to identify the structural difference and then validate the detector by residue substitution^14,40^. To identify the detector of DNA-flexibility in cGAS, we computationally generated a superimposition model to reveal the structural difference(s) among apo- 16 bp, 18 bp and 39 bp DNA–cGAS atomic models. Notably, computational superimposition may not represent biochemically relevant conformations of binding compared to the structural model they compare to, although DNA-flexibility is highly associated with DNA-length^11^. Despite the molecular dynamics simulations suggested that DNA-flexibility might be associated with DNA-length in the above atomic models, more work is still needed to determine whether these models are truly different in flexibility. Second, R222 substitution reduced the cGAS inhibition by nucleosome and led to constant cGAS activation in the cellular context^5,42^. As a result, we failed to build the R222A mouse model to evaluate the role of R222 in vivo. Third, although we show a role of R222 in the DNA-flexibility detection, we did not exclude the possibility that other residue(s) might contribute to the detection particularly in a species-specific manner. Fourth, because the mechanism for high dose IR such as releasing of intracellular contents to mount AIS is well documented, this study was focused on the relative low dose IR. Fifth, although cGAS plays a role in AIS against damaged DNA, we did not exclude the potential contribution of other sensor(s) to the detection. Sixth, the micronucleus generation is mitosis-dependent and most in vivo cells are at G1 or G0 phase, suggesting a less contribution of micronuclei to the AIS mounted by the first IR fraction. But the micronucleus generation and AIS might be overlapped in the context of consecutive daily therapies. Hence, the micronuclei might contribute to AIS in the consecutive daily therapies (particularly > 3 d). Despite of these limitations, our study still showed a cGAS-mediated surveillance of DNA-flexibility.

## Methods

### Cell culture

MC38 cells were maintained in Dulbecco’s modified Eagle’s medium (DMEM) containing 10% foetal bovine serum (FBS), 100 U/mL penicillin and streptomycin at 37 °C under a humidified atmosphere of 5% CO_2_. THP-1 cells were maintained in RPMI-1640 medium (Thermo Fisher Scientific Inc., Waltham, MA, USA) containing 10% FBS. Differentiation of THP-1 cells was induced by the treatment with 0.1 mM PMA (phorbol 12-myristate 13-acetate) for 24 h. MEF cells were prepared and maintained as previously described^15,56^. DNA transfections were performed using Lipofectamine 2000 (Thermo Fisher Scientific Inc., Waltham, MA, USA) according to the manufacturer’s instructions.

### Reagents

Anti-Ifnb1 (D2J1D) (Cat. number: 97450), anti-Actin (Cat. number: 3700), anti-mouse cGAS (Cat. number: 31659) and anti-BrdU (Cat. number: 5292) antibodies were from Cell Signaling (Beverly, MA, USA). Anti-cyclobutane pyrimidine dimers (CPDs) (Cat. number: CAC-NM-DND-001) and anti-Ctla4 (mAb Clone: 9H10) (Cat. number: BE0131) antibodies were from Cosmo Bio (Carlsbad, CA, USA) and Bio X Cell (Lebanon, NH, USA), respectively. Anti-CD8 antibody (Cat. number: ab217344) was from Abcam (Waltham, MA, USA).

Full length human cGAS (NM_138441.3) sequence was cloned into the Nco I and Sal I sites of pET-28a vector (Novogen Limited, Hornsby Westfield, NSW, Australia) to build pET-28a-cGAS (WT) plasmid for the expression of cGAS protein. Then human cGAS amino acid 1-160 and 161-522 sequences were cloned into the pET28a vector to build pET-28a-cGAS-N and pET-28a-cGAS-C truncated mutant plasmids, respectively. Full length mouse cGAS (NM_173386.5) was cloned into the Nhe I and Xho I sites of pET28a vector to build pET-28a-mcGAS (WT) plasmids. Then mcGAS amino acid 147-507 sequence was cloned into the Nhe I and Xho I sites of pET28a vector to build pET-28a-mcGAS-C (WT-C) truncated mutant plasmid. Residue substitution mutant plasmids were generated by site mutation. Mouse wildtype (WT) cGAS sequence (NM_173386.5) was also cloned into the Nhe I and Xba I sites of pTRE-Tight vector (Clontech, Mountain View, CA, USA) to build pTRE-WT cGAS plasmid for Tet-On advanced inducible gene expression. R222E and R222A cGAS sequences also cloned into the Nhe I and Xba I sites of pTRE-Tight vector to build pTRE-R222E cGAS and pTRE-R222A cGAS plasmids, respectively.

ATP (Cat. number: R0441) and GTP (Cat. number: R046) were from ThermoFisher Scientific (Waltham, MA, USA). PMA (Cat. number: P8139), UCN-01 (Cat. number: F2773), HT-DNA (Cat. number: D6898) and RU.521 (Cat. number: SML2347) were from Sigma-Aldrich (St. Louis, MS, USA). 2’3’-cGAMP (Cat. number: tlrl-nacga23) and niraparib (Cat. number: HY-10619) were from InvivoGen (San Diego, CA, USA) and MedChem Express (Shanghai, Shanghai, China), respectively. Nucleasome protein (Cat. number: 52015) was from BPS bioscience (San Diego, CA, USA). Nt.BspQI (Cat. number: R0644S) was from New England BioLabs (Beijing, PRC).

### DNA looping assays

DNA looping assays were performed as previous described^33^. Briefly, 5’-CAGAATCCGT-3’ and its complementary sequence were used as the 10 nucleotide (nt) overhangs for all DNAs. Cy3 donor and Cy5 acceptor fluorophores were attached to the 5’ ends of DNA strands with overhang (Supplementary Data 1). A biotin was attached to a thymine base through a 5-carbon linker (Supplementary Data 1). For the Ctrl and damaged 80 bp DNA set, the 10 nt overhang was directly attached to the 5’ end of the Ctrl and damaged 80 bp DNAs, resulting in 90 bp circular size. The circular size is the circumference of the DNA circle formed after looping and is the sum of the dsDNA length and the overhang length. For the E8 and E8A26 DNA set, DNA looping assays were performed as previously described^33^. For the 50 bp seq1–10 DNA set, 20 nt DNA (10 nt overhang and 10 nt random sequence DNA) was attached to the each 5’ end of the 50 bp seq1–10 DNAs, resulting in 80 bp circular size.

### Expression and purification of cGAS proteins

Briefly, *E. coli* strain, BL21 (DE3), was separately transformed with plasmids encoding the proteins. Then, the cGAS proteins were expressed and purified as previously described^1^. The *E. coli* cells were grown at 37 °C, until an OD_600_ of 0.6 was reached. The temperature was then reduced to 20 °C, and the cells were induced with 0.4 mM isopropyl β-D-1-thiogalactopyranoside (IPTG) for 24 h at 20˚C. The resultant cells were harvested by centrifugation at 5000 x *g* for 15 min and washed twice with cold phosphate-buffered saline (PBS). The collected cells were broken by ultrasonic wave and centrifugated at 24,000 x *g* for 30 min to remove unbroken cells and debris. The soluble fraction was incubated by Ni Sepharose 6 Fast Flow (Cytiva), washed with binding buffer (20 mM Tris-HCl, pH 7.5, 150 mM NaCl, and 30 mM imidazole), and eluted with elution buffer (20 mM Tris-HCl, pH 7.5, 150 mM NaCl, and 300 mM imidazole) as previously described^1^. Concentration of resultant proteins and buffer exchange were performed using an Amicon Ultrafree centrifugal filter (Millipore) with a cutoff of 10 kDa. The proteins were labelled with Alexa Fluor™ 488 using the Alexa Fluor™ 488 Protein Labelling Kit (ThermoFisher, Waltham, MA, USA). The estimated degree of labeling was 2 mol of Alexa Fluor 488 per mol of protein.

### EMSA, MST and MARTFQ assays

EMSA was performed as previously described^57^. For most experiments, 50 nM Cy3-labelled DNA was mixed with a serial dilution of the noted proteins in the buffer containing 20 mM Tris-HCl (pH 7.5) and 150 mM NaCl. After incubation at 37 °C for 30 min, the mixtures were analysed on 1.5% agarose gel. Images were acquired by ChemiDoc XRS + (Bio-rad) and analysed by Image J. The Kd was calculated with a binding equilibrium equation using GraphPad Prism. For 0N0 experiment, 44 bp noted DNA, cGAS and nucleosome (0N0) mixture were performed as previously described^18^.

MST was performed as previously described^58^. Briefly, 50 nM Cy5-labelled DNA was mixed with a serial dilution of the cGAS protein in the buffer containing 20 mM Tris-HCl (pH 7.5) and 150 mM NaCl at room temperature. Then, the thermophoretic movement of DNA with protein was analysed by Monollith NT.115 instrument (Nanotemper Technologies). And the Kd of the noted DNA was calculated by GraphPad Prism.

MARTFQ assay was performed as described in our previous study^58^. Briefly, 50 nM of cGAS protein was mixed with a serial dilution of DNA as noted. After incubation for 5 min, the samples were subjected to fluorescence analyses based on 348 nm intrinsic protein fluorescence excited by 280 nm light using Fluorescence Spectrophotometer F-7000 (Hitachi high Technologies, Tokyo, Japan). Scan speed was set on 1200 nm/min. EX Slit and EM Slit were set to 5.0 nm. The Kd dissociation constant was estimated according to the modified Stern-Volmer equation: RF0/ΔRF = (Kd/*f*) × (1/[*Q*]) + (1/*f*), where ∆RF is equal to RF0 (the protein fluorescence intensity in the absence of metabolite) - RF (the intensity in the presence of metabolite); *f* is the fractional maximum fluorescence intensity of protein; Kd is 1/quenching constant^59^; and [*Q*] is the concentration of DNA.

### cGAS–DNA condensation assays

In vitro cGAS–DNA condensation analyses were performed as previously described^3^. cGAS protein (5% AF488-labelled) was mixed with Cy3-labelled DNA in a buffer containing 20 mM Tris-HCl (pH 7.5) and 100 mM NaCl. The mixtures were then incubated at 37 °C for 10 min and the turbidities were assessed at 340 nm by NanoDrop 2000C (Thermo Scientific, Waltham, MA, USA). Fluorescent images of the mixtures incubated for the indicated times were captured by fluorescence microscopy (Olympus IX81, Japan).

### Quantification of cGAMP by LC-MS/MS

The samples were prepared as previously described^58,60^. The resultant samples were separated using the ACQUITY UPLC system (Waters) followed by MS detection using a Qtrap5500 triple quadrupole mass spectrometer (ABSciex) in positive electrospray ionisation mode. Multiple-reaction-monitoring (MRM) mode was used to monitor cGAMP parent ion to product ions: m/z 675 to 136 (collision energy (CE) 45 V; declustering potential (DP) 70 V). The ionspray voltage was maintained at 5500 V. The turbo gas temperature was set at 550 °C. Curtain Gas (CUR), Ion Source Gas1 (GS1) and GS2 are set at 20 psi, 40 psi and 60 psi, respectively. Both Q1 and Q3 quadrupoles were maintained at unit resolution. The mobile phases (delivered at 0.45 mL/min) consisted of H_2_O (containing 20 mM NH_4_Ac and 0.05% HAc) for A and methanol (containing 20 mM NH_4_Ac and 0.05% HAc) for B. LC isocratic elution (50% A and 50% B) was performed at a stop time of 5 min. The Analyst software for Windows (Applied Biosystems, Darmstadt, Germany) (version 1.6.2) was used for data acquisition and MS peak area quantification.

### cGAS activity assays

cGAS activity assays were performed as previous described^3^. Briefly, cGAS proteins at the noted concentrations were mixed with free DNA or NCP DNA in the reaction buffer containing 20 mM Tris-HCl (pH 7.5), 1.5 mM MgCl_2_, 2 mM ATP and 2 mM GTP. After incubation at 37 °C for 2 h, the mixtures were diluted by 5-fold and centrifuged at 16,000 *g* for 10 min. The resultant supernatants were then analysed by liquid chromatography with tandem LC–MS/MS as described above. For the inhibition of cGAS by nucleosome (0N0), 50 nM full length cGAS was mixed with serial dilutions of nucleosomes in the reaction buffer containing 10 mM HEPES (pH 8.0), 100 mM NaCl and 1.5 mM MgCl_2_, 100 nM noted DNA, 1 mM ATP and 1 mM GTP. After incubation at 37 °C for 2 h, resultant cGAMP was quantified by LC-MS/MS. The IC_50_ (half maximal inhibitory concentration) was calculated using GraphPad Prism.

### Real-time quantitative reverse transcription PCR (qRT-PCR)

QRT-PCR was performed as described in our previous study^58^. Briefly, RNA was extracted using the RNeasy Mini Kit (QIAGEN, Hilden, Germany). One microgram of total RNA was used for reverse transcription using the PrimeScript RT Reagent Kit (TaKaRa, DaLian, Liaoning, China). qRT‒PCR was performed using the SYBR PrimeScript PCR Kit II (TaKaRa, DaLian, Liaoning, China) by Mx3005P™ Real-Time PCR System. The primers used for qRT‒PCR are described in Supplementary Data 1.

### Elisa assays

According to the manufacturer’s instructions, Elisa assays were performed using Ifnb ELISA kit (Cat. Number: 439407, Biolegend, San Diego, CA, USA) or CXCL10 ELISA Kit (Cat. Number: E4827-100, Biovision, Milpitas, CA, USA).

### Circular dichroism assay

Circular dichroism spectra (190 nm to 260 nm) of 0.5 mg/mL cGAS proteins were measured in a buffer containing 10 mM HEPES (pH 7.5) and 100 mM NaCl by a Chirascan spectrometer (Applied Photophysics Ltd., U.K) at room temperature^42^.

### Dynamic light scattering assay

Dynamic light scattering (DLS) analyses were performed as previously described^61^. Briefly, 1 mg/mL cGAS proteins in a DLS buffer (20 mM Tris-HCl pH 7.5, 100 mM NaCl and 1 mM dithiothreitol) were centrifuged at 5000 × *g* for 10 min and then analysed by SZ-100 Nanoparticle Size and Zeta Potential Analyzer (Horiba, Japan).

### SDS and Native PAGE analyses

Sodium dodecyl sulfate (SDS) and Native polyacrylamide gel electrophoresis (PAGE) gel analyses were used to examine cGAS protein dimerisation as previously described^62^. For SDS PAGE analysis, full-length WT and R222A mcGAS proteins were incubated with a loading buffer with or without β-mercaptoethanol at 37 °C. For Native PAGE analysis, full-length WT and R222A mcGAS proteins were incubated with a loading buffer without β-mercaptoethanol. The protein bands were visualized by Coomassie blue staining.

### Tet-On inducible cGAS gene expression

Tet-On induction of cGAS gene expression was performed according to the manufacturer’s instructions (Clontech, Mountain View, CA, USA). Briefly, the immortal cGAS KO MEF cells were transfected with pTet-On Advanced plasmid and selected by G418. The resultant cell line form single clone was transfected with pTRE-WT cGAS, pTRE-R222E cGAS or pTRE-R222A cGAS plasmid. After a puromycin selection, the stable pTRE-WT cGAS, pTRE-R222E cGAS and pTRE-R222A cGAS cells with the similar inducible potentials were used for the further study. For the doxycycline (Dox) induction, the above cells were treated with 1 ng/mL, 10 ng/mL or 100 ng/mL doxycycline as noted for 24 h.

### DNA–cGAS structural analyses

The superimposition atomic models were generated computationally using PyMOL Molecular Graphic Systems (version 2.5.2) using the default parameters. Briefly, the structural data for apo-, 16 bp, 18 bp and 39 bp (4K8V, 4K96, 4LEZ, 5N6I) DNA–cGAS complexes were derived from the Protein Data Bank database (https://www.rcsb.org). PyMOL was used to align 18 bp DNA–cGAS atomic mode to 16 bp DNA–cGAS atomic model. Then, 39 bp DNA–cGAS atomic model was aligned to the 18–16 superimposition atomic model using PyMOL. Finally, apo cGAS atomic model was aligned to the resultant 39–18–16 superimposition atomic model.

### Estimation of conformational energy

Conformational energetic changes of R as a free anima acid are calculated by Hyperchem using default parameters.

### Calculation of free energy of protein–DNA binding

The free energy (ΔG) of protein–DNA binding in 16 bp, 18 bp and 39 bp DNA–cGAS atomic models were assessed using gmx_MMPBSA (version 1.5.6)^63^, which is based on Molecular mechanics/Poisson-Boltzmann surface area (MMPBSA) analysis. Briefly, the data of 16 bp, 18 bp and 39 bp DNA–cGAS atomic models were derived from the Protein Data Bank database (https://www.rcsb.org). The input files were prepared according to the gmx_MMPBSA instructions for protein-DNA binding free energy calculations (https://valdes-tresanco-ms.github.io/gmx_MMPBSA/dev/examples/Protein_DNA/). cGAS and DNA were set as receptor and ligand, respectively. The calculations were performed using the default parameters. 16 bp and 18 bp DNA bind cGAS to form cGAS_2_–DNA_2_ complex, while 39 bp DNA binds cGAS to form cGAS_4_–DNA_2_ complex. To normalise the structural difference, the values of ΔG, H and –TS from 39 bp DNA–cGAS complex were divided by 2. The energetic models were visualized by PyMOL (version 2.5.2).

### Dot blotting analyses of CPD

Dot blotting analyses of CPD were performed as described in our studies^58^. Total genomic DNA was extracted using the PureLink™ Genomic DNA Mini Kit (ThermoFisher, Waltham, MA, USA) according to manufacturer’s instructions. After boiling for 5 min to denature DNA, 250 ng of DNA were spotted on Hybond-N + membrane and cross-linked by UV using Stratalinker 2400 (Stratagene, La Jolla, CA, USA). The membrane was blocked in 5% BSA and then incubated with anti-CPD monoclonal antibody for 1 h. After washing with the phosphate-buffered saline (PBS) buffer containing 0.2% Tween 20 (PBS-T), the membrane was incubated a peroxidase-labelled anti-mouse monoclonal antibody (Cell Signaling, Beverly, MA, USA) for 1 h, and the chromogenic reaction was performed using an ECL chemiluminescence kit (ThermoFisher Scientific, Waltham, MA, USA). After washing with PBS-T buffer, the membrane was stained by methylene blue for 20 min and imaged.

### Histologic analysis

For Hematoxylin and Eosin (H&E), the skin sections were stained with H&E according to standard protocols using commercially available reagents. Immunofluorescent analyses were performed as previously described^64^. Skin, intestine and tumour tissues were fixed with 4% paraformaldehyde and embedded in paraffin. The skin and intestine sections (4 μm) were incubated with anti-Ifnb and anti-BrdU antibodies as noted at 4 °C for overnight, while the tumour sections (4 μm) were incubated with anti-CD8 antibody at 4 °C for overnight. After washing, the resultant sections were incubated with the appropriate secondary antibodies, and mounted with mounting medium supplemented with 4’,6-diamidino-2-phenylindole (DAPI) (Vector Laboratories, Burlingame, CA, USA). Fluorescent images of the sections were captured by fluorescence microscopy (Olympus IX81, Japan).

### Preparation of nucleosomal DNA

0N0 and 4 × NCP were prepared as previously described^18^. Previously described 4 × nucleosome core particles (NCPs) positioned by widom-601 sequences^65^ spaced by 44 bp linkers (4 × NCP) with Nt.BspQI cleaving sites (Supplementary Data 1) were applied to generate nick 4 × NCP. To prepare the nucleosome, the human H2A, H2B, H3.3 and H4 histone proteins were mixed at a 1.2:1.2:1:1 molar ratio in unfolding buffer containing 25 mM Tris-HCl (pH 7.5), 7 M GdmCl and 1 mM dithiothreitol (DTT)^18^. Refolding was achieved by dialysis against a buffer containing 25 mM Tris-HCl (pH 7.5), 2 M NaCl and 1 mM DTT, followed by purification with size exclusion chromatography. Then, the nucleosome was mixed with 0N0 or 4 × NCP sequence DNA (Supplementary Data 1) in a buffer containing 25 mM Tris-HCl (pH 7.5), 2 M NaCl and 0.25 mM DTT. Final assembly of the nucleosomal DNA complex was achieved by dialysis against a low-salt buffer containing 25 mM Tris-HCl (pH 7.5), 50 mM NaCl and 0.25 mM DTT for overnight. Following the nickase treatment, the nick 0N0 and 4 × NCP were purified by size exclusion chromatography and used for further cGAS activity assays.

### Animals

The animal handling procedures were approved by the Animal Care and Use Committee of DaLian Medical University. To create a cGAS knockout mouse model (C57BL/6) by CRISPR/Cas-mediated genome, we designed a construct resulting in the deletion of 2–4 exons of the cGAS coding sequence. Cas9 mRNA and sgRNA generated by in vitro transcription were then injected into fertilised eggs for KO mouse productions. The founders were genotyped by PCR (F1: 5’-TATGTACAGGAACCCGTGCAG-3’; R1: 5’-CTTAACCACTGAGCCATCTCTAG-3’) followed by DNA sequencing analysis. The positive founders were bred to the next generation (F1) and subsequently genotyped by PCR (F2: 5’-TTCACTAAATAGACCAAGCTGCTG-3’; R2: 5’-ATGACTCAGCGGATTTCCTCG-3’), DNA sequencing and immunoblotting analyses. All mice were housed in a specific pathogen-free facility at 22 ± 2 °C under a cycle of 12 h light (7:00 am light on) and 12 h dark (7:00 pm light off). For Skin experiment, mice were treated with 150 μg/cm^2^ RU.521, 5 μg/cm^2^ niraparib, 5 μg/cm^2^ UNC-01 or vehicle (H_2_O, ethanol and dimethylsulphoxide) for 1 h.

Tumour challenge and treatment were performed as previously described^27,50^. Briefly, female 5–8-week-old mice were injected subcutaneously (s.c.) with 5 × 10^5^ MC38 cells in the right flank to build single neoplastic lesion, or 5 × 10^5^ MC38 cells in the right flank and then 5 × 10^5^ MC38 cells contralaterally 2 d later to build double neoplastic lesions. On day 10, mice were randomly grouped as noted. For IR experiment, one or three focal fractions of 8 Gy using the X-RAD320 Small Animal Irradiator (Precision X-Ray, North Branford, CT, USA) were applied to irradiate the mice pretreated with noted inhibitor for 3 h. For cGAS inhibition experiment, RU.521 was injected i.p. (5 mg/kg) at 3 h prior to IR on the noted day. For niraparib experiment, mice were treated by oral gavage with niraparib (4 mg/kg) at 3 h prior to IR as previously described^66^. Vehicle was used as control. For ICB experiment, CTLA-4–blocking mAb 9H10 was injected i.p. (10 mg/kg) on D12, D15, and D18. CD8 + T cells were assessed as previously described^27^. Tumour size was measured daily by caliper and tumour volumes were calculated using the formula: 0.52 × *L* × *W*^2^, where *L* was the longest diameter and *W* was the shortest diameter. Once the longest diameter of tumour was above 1.5 cm, mice were sacrificed according to the handling procedures of the Animal Care and Use Committee.

### Statistics and reproducibility

All statistical tests, comparisons, replications and sample sizes are included in figure legends. All data are presented as mean values ± standard deviations (SDs), and error bars indicate standard deviations. All experiments were replicated at least three times, and all attempts at replication were successful. Unless specifically noted, statistical analyses were performed using unpaired two-tailed Student’s *t* test without adjustment for multiple comparisons. For the Kaplan‒Meier analyses, log-rank test was used to calculate the *p* values. For the correlation analyses in Fig. 3, two-sided Spearman and two-sided Pearson analyses were performed as noted. In all cases, ***p < 0.001, **p < 0.01, *p < 0.05, N.S. = not statistically significant (p > 0.05). All statistical analyses were performed using Prism 8 (GraphPad Software).

### Reporting summary

Further information on research design is available in the Nature Portfolio Reporting Summary linked to this article.

## Supplementary information

Supplementary Information

Description of additional Supplementary File

Supplementary Data 1

Reporting Summary

## Data Availability

The data supporting the findings of this study are available within the article and its supplementary figures, and from the corresponding authors upon request. The structural data for apo- (4K8V), 16 bp (4K96), 18 bp (4LEZ) and 39 bp (5N6I) DNA–cGAS complexes were obtained from the Protein Data Bank database (https://www.rcsb.org). Source data are provided with this paper.

## Source data

Source Data
