# Supplementary material for: DNA mechanical flexibility controls DNA potential to activate cGAS-mediated immune surveillance
Source: Nat Commun. 2022 Nov 19;13:7107. doi: 10.1038/s41467-022-34858-6 (PMC9675814; doi:10.1038/s41467-022-34858-6)
Supplement: Supplementary file 1 — Supplementary Information [file 41467_2022_34858_MOESM1_ESM.pdf]

DNA mechanical flexibility controls DNA potential to activate cGAS-mediated immune surveillance

Wang *et al*.

Supplementary Information

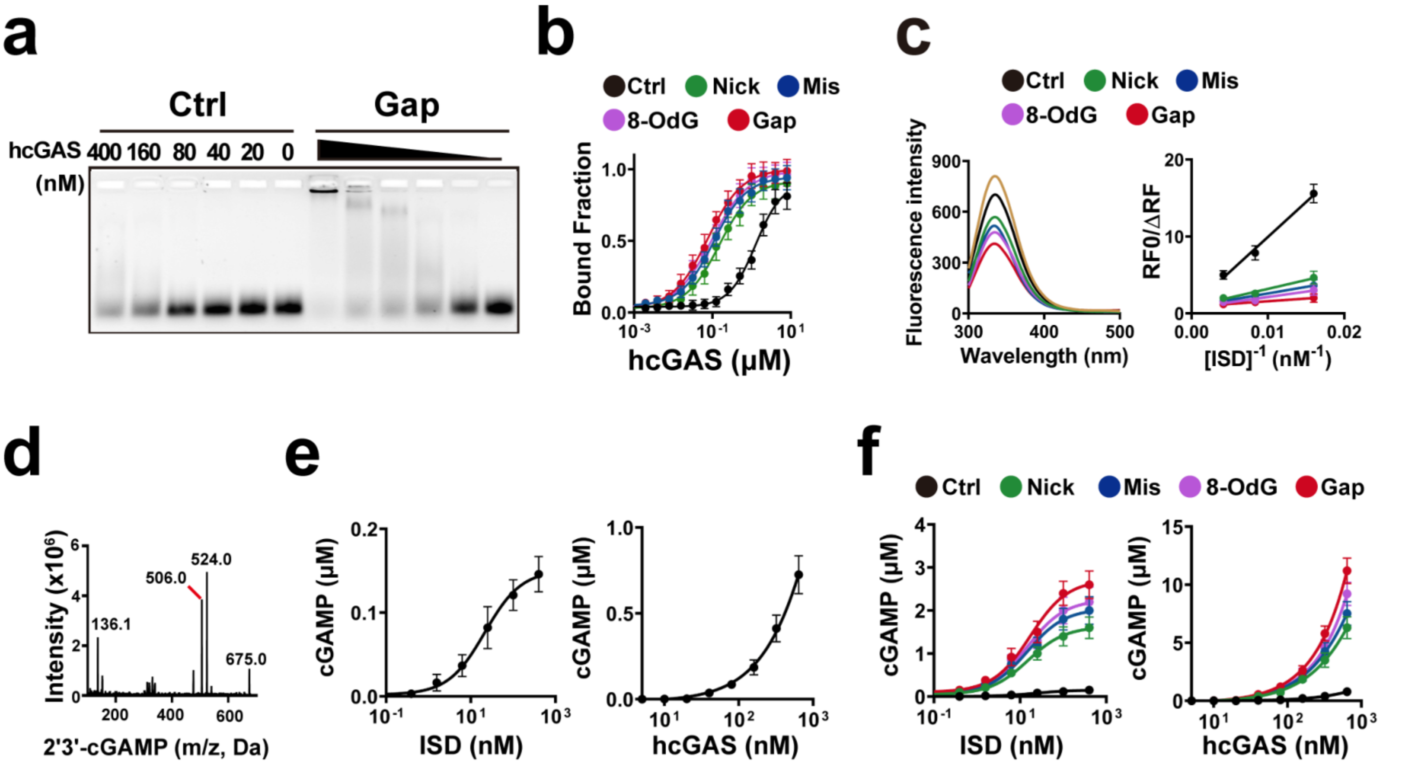

Supplementary Fig. 1 Damaged-increased DNA-flexibility elevates the DNA potential to bind and activate cGAS in vitro. a, EMSA assays of 50 nM 44-bp Ctrl or gap ISD binding to serial dilutions of hcGAS protein. **b,** MST assays of cGAS binding to 50 nM of Cy5-labelled ISD. The Kds were from the plot of bound fraction as a function of the cGAS concentration. Kds of cGAS for the ISD--Ctrl: 1269 ± 308.5 nM, Nick: 150.5 ± 42.6 nM, Mis: 108.4 ± 26.3 nM, 8-OdG: 97.9 ± 25.2 nM and Gap: 75.8 ± 18.6 nM. Mean ± SD. n = 3 independent experiments. **c,** Left: Quenching of intrinsic protein fluorescence of 50 nM cGAS by 100 nM noted ISD. Right: The modified Stern-Volmer curves to estimate Kds were built as described in Methods. Kds of cGAS for the ISD-Ctrl: 1101.1 ± 289.6 nM, Nick: 257.7 ± 65.5 nM, Mis: 194.9 ± 51.3 nM, 8-OdG: 193.8 ± 45.7 nM and Gap: 79.4 ± 20.1 nM. Mean ± SD. n = 3 independent experiments. **d,** 2'3'-cGAMP ion mass transition for MS analysis. **e,** Left: cGAMP produced by 100 nM cGAS in serial dilutions of Ctrl-ISD for 2 h. 100 nM cGAS protein was mixed with serial dilutions of Ctrl-ISD. After incubation for 2 h, the produced cGAMP levels in the mixtures were assessed by MS analysis. The curve was built by plotting the cGAMP production versus the Ctrl-ISD concentration. Right: cGAMP produced by serial dilutions of cGAS in 100 nM Ctrl-ISD for 2 h. Mean ± SD. n = 3 biologically independent samples. **f,** Left: cGAMP produced by 100 nM cGAS in serial dilutions of noted ISD for 2 h. Right: cGAMP produced by serial dilutions of cGAS in 100 nM noted ISD for 2 h. Mean ± SD. n = 3 biologically independent samples. Source data are provided as a Source Data file.

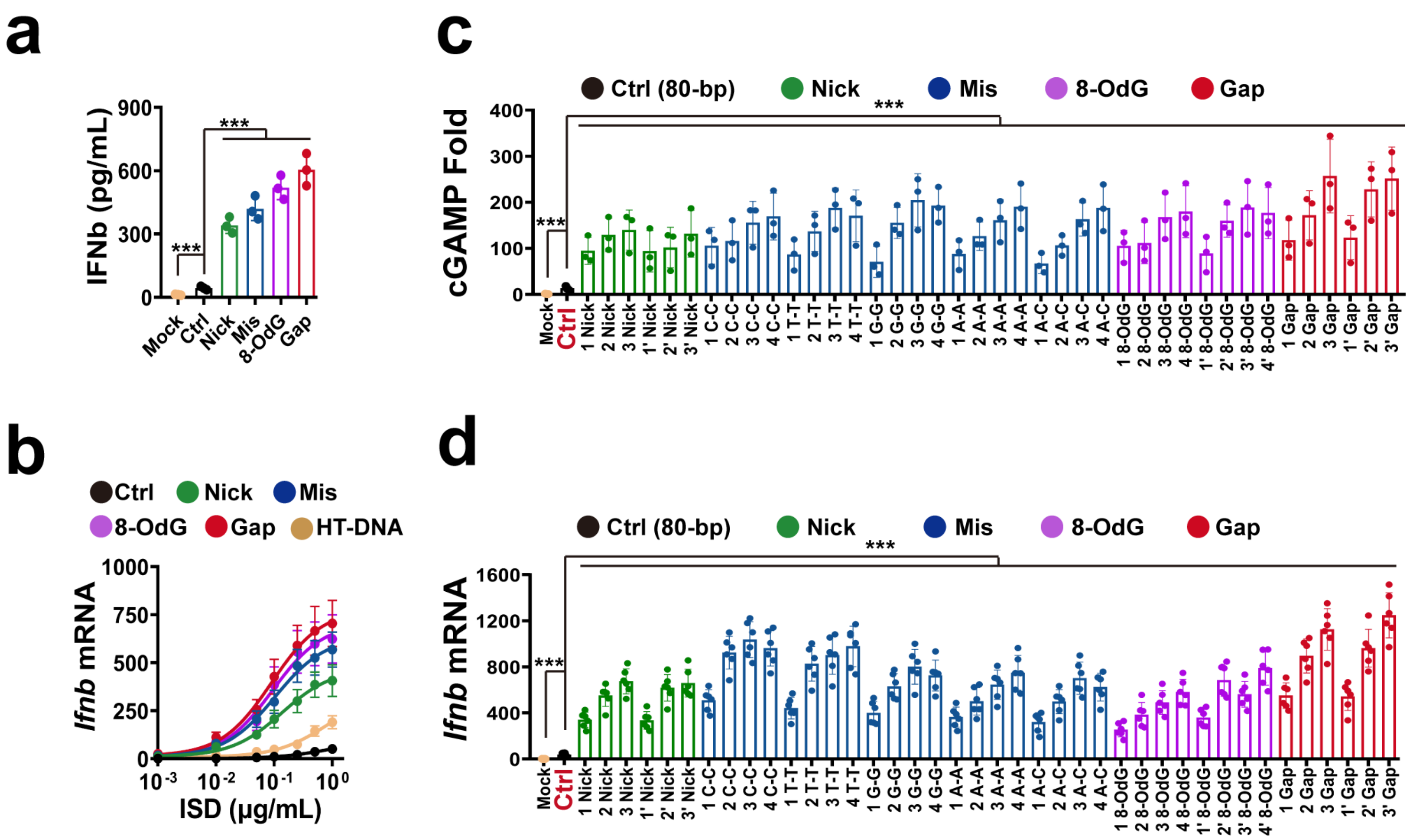

Supplementary Fig. 2 Damaged-increased DNA-flexibility elevates the intracellular DNA potential to bind and activate cGAS. a, Elisa analyses of the secretion of Ifnb protein from the MEF cells transfected with 100 ng/mL noted ISD for 24 h. Mean ± SD. n = 3 biologically independent samples. **b,** Ifnb mRNA levels in the MEF cells transfected with serial dilutions of 44-bp noted ISD or herring testis DNA (HT-DNA) for 2 h. Mean ± SD. n = 3 biologically independent samples. **c,d,** cGAMP (**c**) and Ifnb mRNA (**d**) levels in the cells transfected with 100 ng/mL noted ISD for 2 h. 1–4: the damage site number (Supplementary Data 1). ’: the damage sites on the complementary strand. Unless specifically noted, the cellular Ifnb mRNA levels were analyzed by real-time quantitative reverse transcription PCR (qRT-PCR). Mean ± SD. cGAMP (**c**): n = 3 biologically independent samples. Ifnb mRNA (**d**): n = 6 biologically independent samples. Source data are provided as a Source Data file.

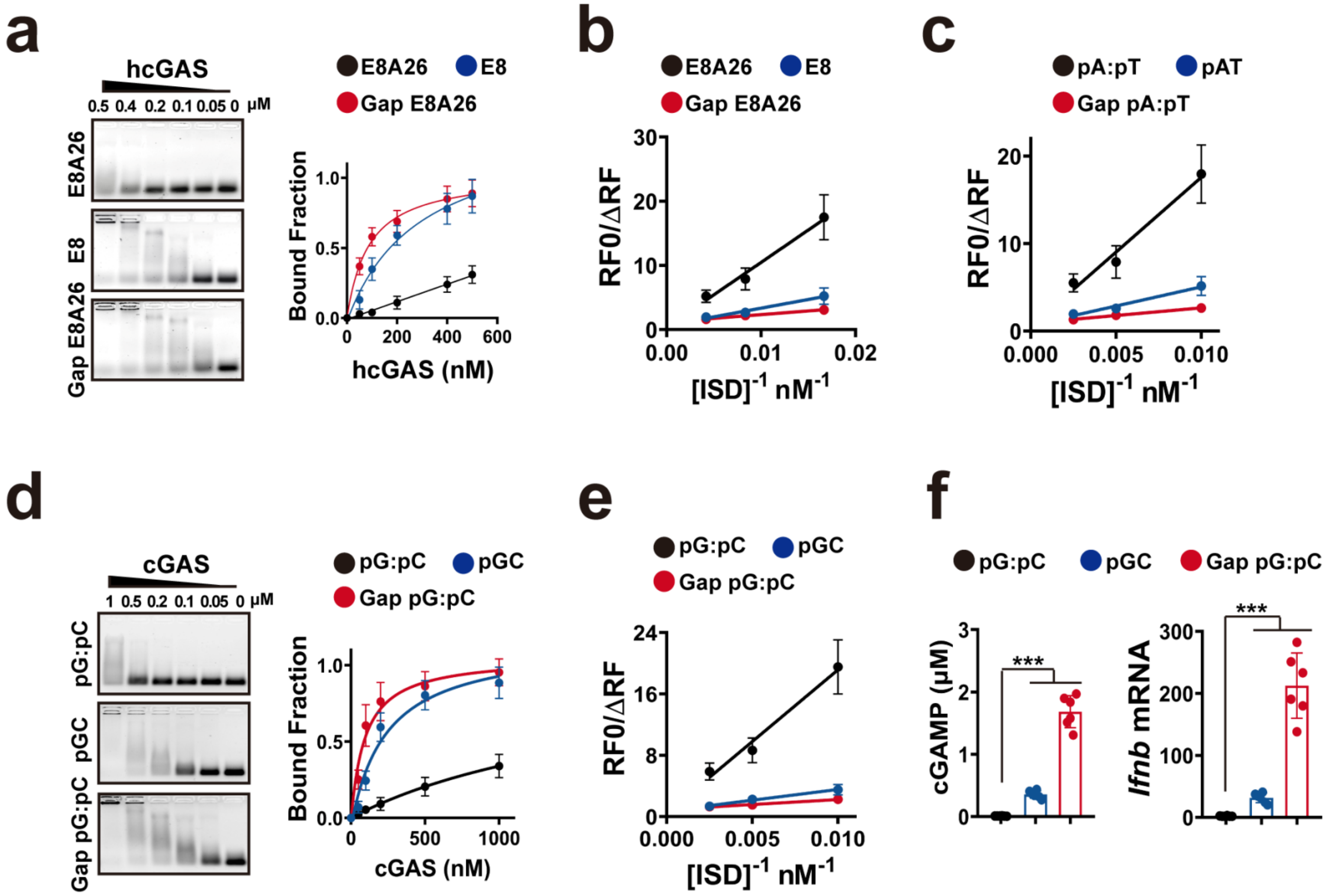

Supplementary Fig. 3 DNA-flexibility regulates the DNA potential to bind and activate cGAS. a, EMSA analyses of 50 nM noted ISDs binding to serial dilutions of cGAS protein. Mean ± SD. n = 3 biologically independent samples. **b,** MARTFQ analyses of the quenching of 50 nM cGAS fluorescence by the noted ISD. Kds of cGAS for E8A26: 2591.9 ± 756.8 nM, E8: 418 ± 85.7 nM, and Gap E8A26: 104.8 ± 19.4 nM. Mean ± SD. n = 3 independent experiments. **c,** MARTFQ analyses of the quenching of 50 nM cGAS fluorescence by the noted ISD. Kds of cGAS for pA:pT: 3461.6 ± 636.9 nM, pAT: 639.93 ± 122.8 nM, and Gap pA:pT: 190.1 ± 35.2 nM. Mean ± SD. n = 3 independent experiments. **d,** EMSA analyses of 50 nM 44-bp noted ISDs binding to serial dilutions of cGAS protein. Mean ± SD. n = 3 independent experiments. **e,** MARTFQ analyses of the quenching of 50 nM cGAS fluorescence by the noted ISD. Kds of cGAS for pG:pC: 4153.2 ± 869.8 nM, pGC: 376.15 ±78.75 nM, and Gap pG:pC: 139.5 ± 24.91 nM. Mean ± SD. n = 3 independent experiments. **f,** Left: in vitro cGAMP produced by 100 nM cGAS in the noted ISD for 2 h. Right: Ifnb mRNA levels in the MEF cells transfected with 100 ng/mL noted ISD for 2 h. Mean ± SD. n = 6 biologically independent samples. Source data are provided as a Source Data file.

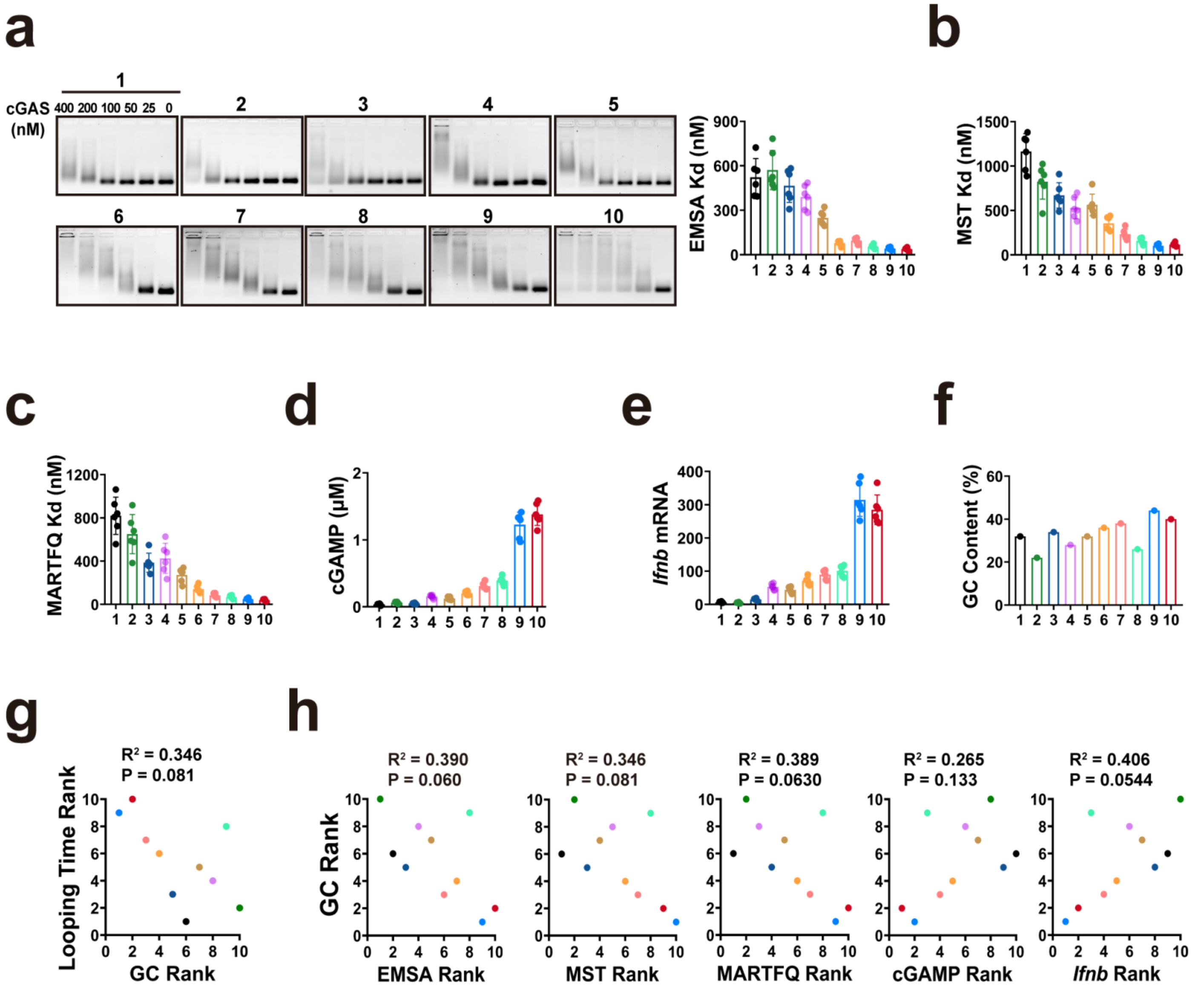

Supplementary Fig. 4 cGAS surveillance of flexibility reveals that DNA sequence controls the cGAS activation. a, EMSA analyses of 50 nM 50-bp noted ISDs (seq1–10) binding to serial dilutions of cGAS protein. Bound fraction was built as a function of the cGAS concentration to calculate Kd of noted ISDs. Mean ± SD. n = 6 biologically independent samples. **b,c,** MST (**b**) and MARTFQ (**c**) Kd of the noted ISDs. Mean ± SD. n = 6 biologically independent samples. **d,** cGAMP produced by 100 nM cGAS in 100 nM noted ISD for 2 h. Mean ± SD. n = 6 biologically independent samples. **e,** Ifnb RNA levels in the MEF cells transfected with 100 ng/mL noted ISD for 2 h. Mean ± SD. n = 6 biologically independent samples. **f,** GC contents of the noted ISDs. **g,** Spearman analyses of the correlation between the DNA looping time and GC content. P and R values were from two-sided spearman analyses. **h,** Spearman analyses of the correlation between the DNA GC content and noted cGAS-binding or cGAS-activating potential. P and R values were from two-sided spearman analyses. Source data are provided as a Source Data file.

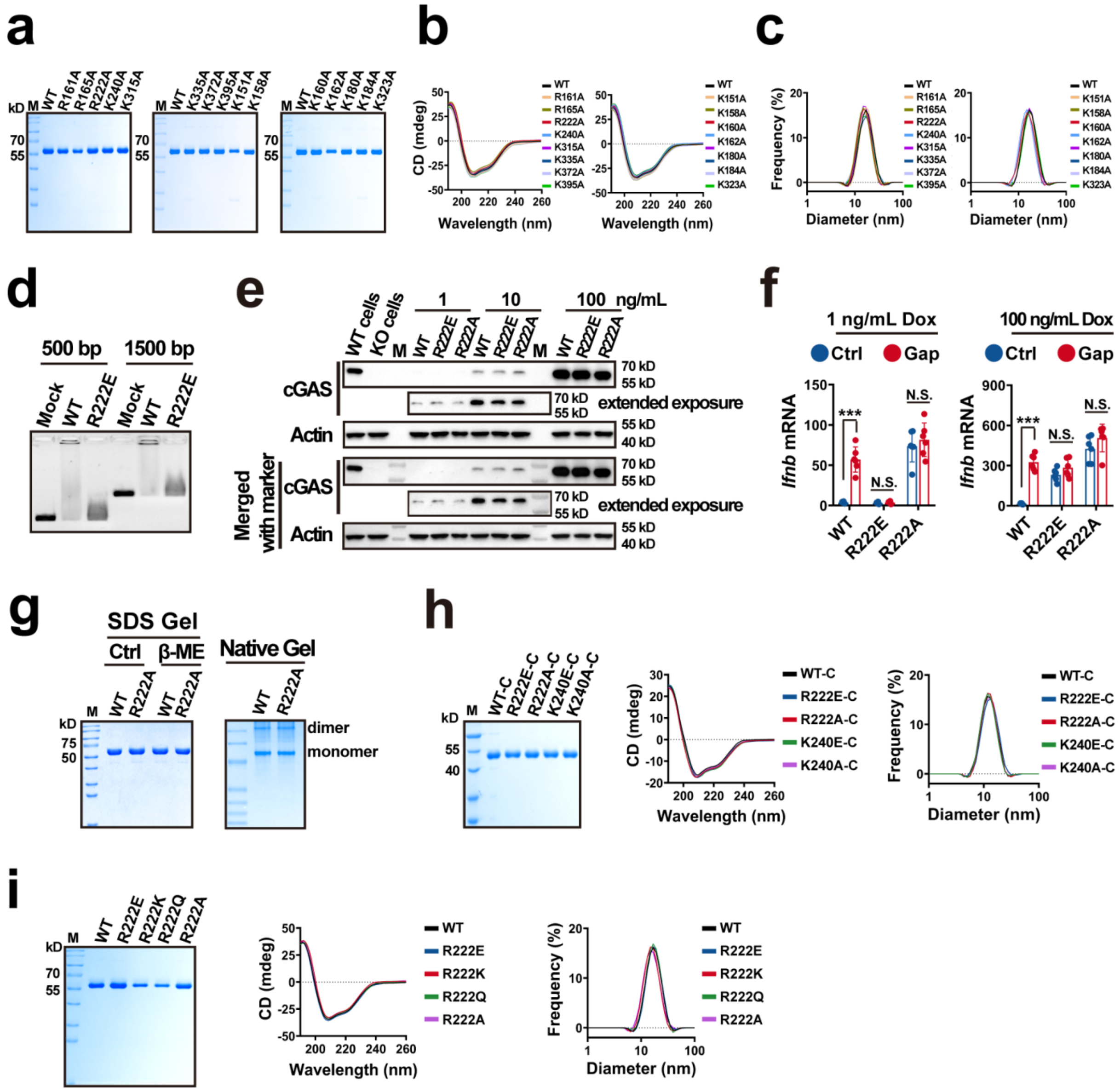

Supplementary Fig. 5 Substitution analyses identified a role of R222 in the DNA-flexibility detection. a, Coomassie staining of purified mutant mouse cGAS (mcGAS) proteins. M: marker; kD: kilodaltons. **b,** Circular dichroism (CD) analyses of mutant mcGAS proteins. mdeg: millidegrees. **c,** Dynamic light scattering (DLS) analyses of noted mcGAS proteins. **d,** EMSA analyses of 50 nM 500- and 1500-bp PCR DNAs binding to WT or R222E mcGAS protein. **e,** Immunoblotting of the lysates from the inducible cells treated with doxycycline at noted concentrations. cGAS KO MEF cells were transfected with pTet-On Advanced plasmid and selected by G418. The resultant cell line was transfected with pTRE- mouse WT cGAS, pTRE-R222E cGAS or pTRE-R222A cGAS plasmid. After a puromycin selection, the stable WT, R222E and R222A cells with the similar inducible potentials were treated with 1 ng/mL, 10 ng/mL or 100 ng/mL doxycycline (Dox) for 24 h. Resultant lysates were subjected to immunoblotting with an anti-mouse cGAS antibody. The lysates from cGAS WT and KO cells were used as controls. Due to the low expression of cGAS in 1 ng/mL Dox treatment, the exposure of a part of the membrane was extended. And the immunoblotting image was merged with the bright filed image of marker to evaluate the molecular weights of cGAS bands. The samples were derived from the same experiment and that gels/blots were processed in parallel. M: marker; kD: kilodaltons. **f,** Ifnb mRNA levels in the inducible cells treated with Dox and then ISD transfection. The inducible cells were treated with 1 ng/mL or 100 ng/mL Dox for 24 h. Then, the cells were transfected with 1000 ng/mL or 100 ng/mL noted ISD for 2 h. Mean ± SD. n = 6 biologically independent samples. **g,** Sodium dodecyl sulfate (SDS) (left) and Native (right) polyacrylamide gel electrophoresis analyses of the noted mcGAS dimerization. β-Me: β-mercaptoethanol; M: marker; kD: kilodaltons. **h,i,** Coomassie staining, CD and DLS analyses of the C-terminal (**h**) and R222 substitution (**i**) mutant mcGAS proteins. M: marker; kD: kilodaltons. Source data are provided as a Source Data file.

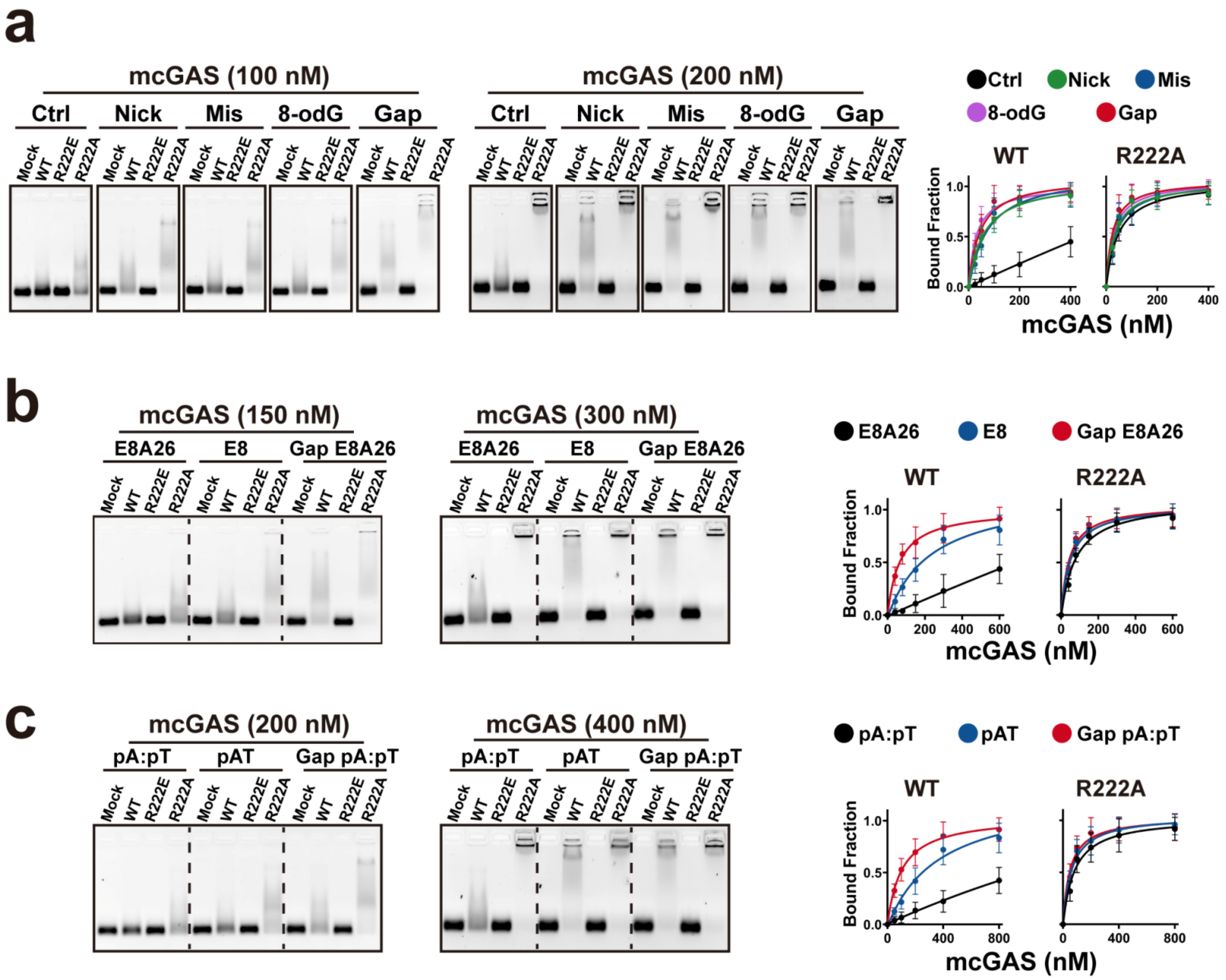

Supplementary Fig. 6 Substitution analyses identified a role of R222 in the DNA-flexibility detection. a,b,c, EMSA analyses of 50 nM damaged (**a**), E8A26/E8 (**b**) or pA:pT/pAT (**c**) ISD binding to the noted mcGAS proteins. Mean ± SD. n = 3 independent experiments. Source data are provided as a Source Data file.

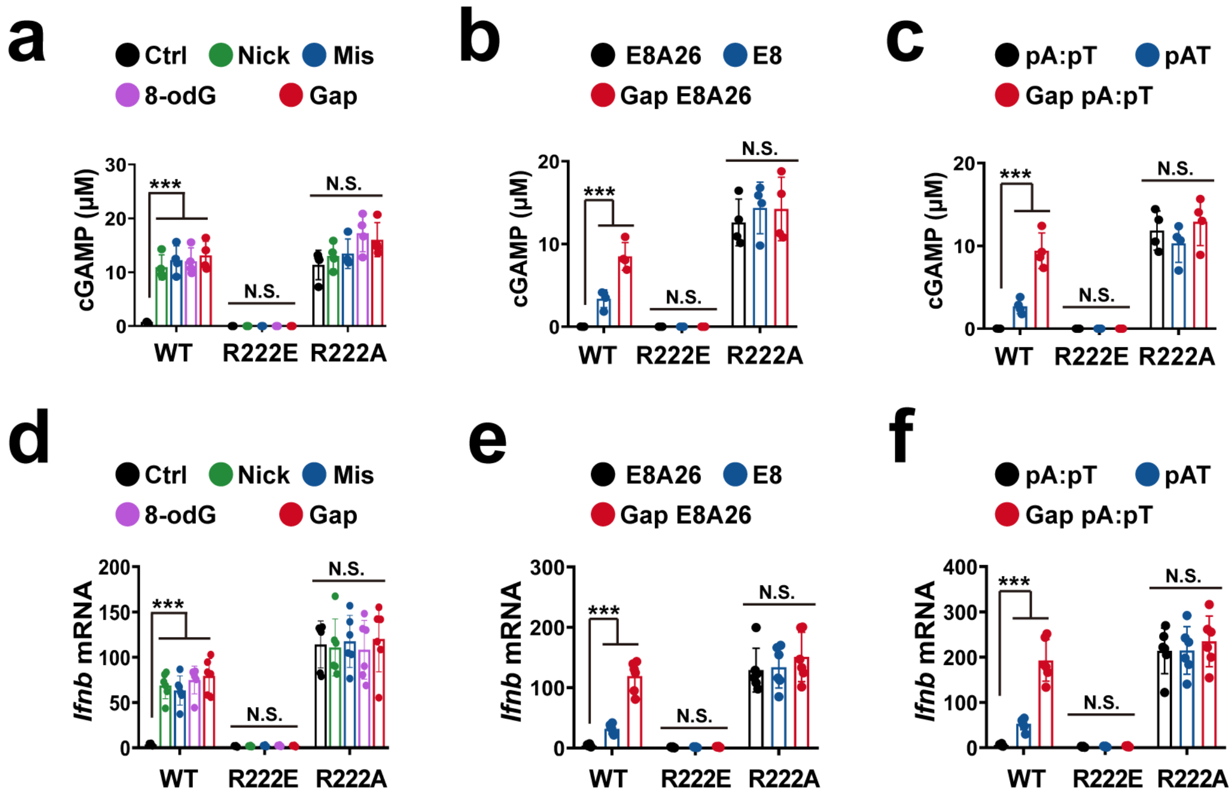

Supplementary Fig. 7 Substitution analyses identified a role of R222 in the DNA-flexibility detection. a,b,c, cGAMP produced by 100 nM noted mcGAS in 100 nM damaged (**a**), E8A26/E8 (**b**) or pA:pT/pAT (**c**) ISD in vitro for 2 h. Mean ± SD. n = 4 biologically independent samples. **d,e,f,** Ifnb mRNA levels in the inducible cells treated with Dox and then ISD transfection. The inducible cells were treated with 1 ng/mL Dox for 24 h. Then, the cells were transfected with 1000 ng/mL damaged (**d**), E8A26/E8 (**e**) or pA:pT/pAT (**f**) ISD. After 2 h, Ifnb mRNA levels in the resultant cells were assessed by qRT-PCR. Mean ± SD. n = 6 biologically independent samples. Source data are provided as a Source Data file.

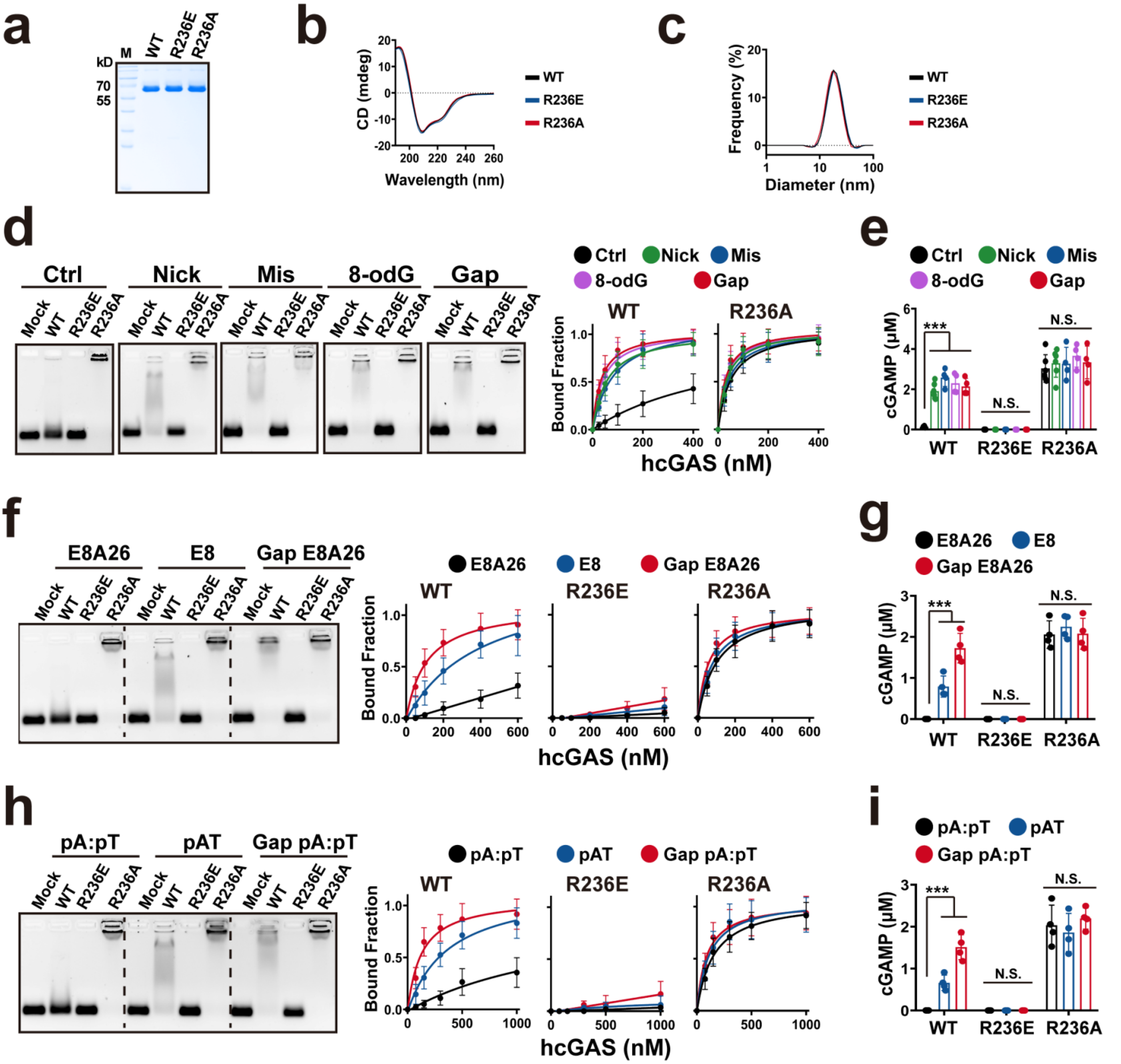

Supplementary Fig. 8 Substitution analyses identified a role of R222 in the DNA-flexibility detection. a,b,c, Coomassie staining (**a**), CD (**b**) and DLS (**c**) analyses of human WT, R236E and R236A cGAS proteins. **d,f,h,** EMSA analyses of 50 nM damaged (**d**), E8A26/E8 (**f**) or pA:pT/pAT (**h**) ISD binding to the noted human hcGAS proteins. Mean ± SD. n = 3 independent experiments. **e,g,i,** cGAMP produced by 100 nM noted mcGAS in 100 nM damaged (**e**), E8A26/E8 (**g**) or pA:pT/pAT (**i**) ISD for 2 h. Mean ± SD. n = 6 biologically independent samples. Source data are provided as a Source Data file.

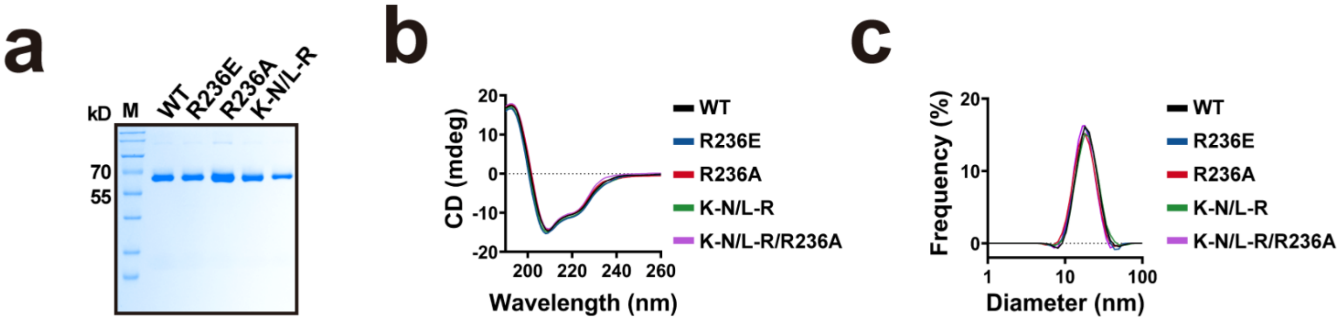

Supplementary Fig. 9 Human R236 substitution relaxes the DNA-length specificity of cGAS. a,b,c, Coomassie staining (**a**), CD (**b**) and DLS (**c**) analyses of noted substitution proteins of human cGAS. Source data are provided as a Source Data file.

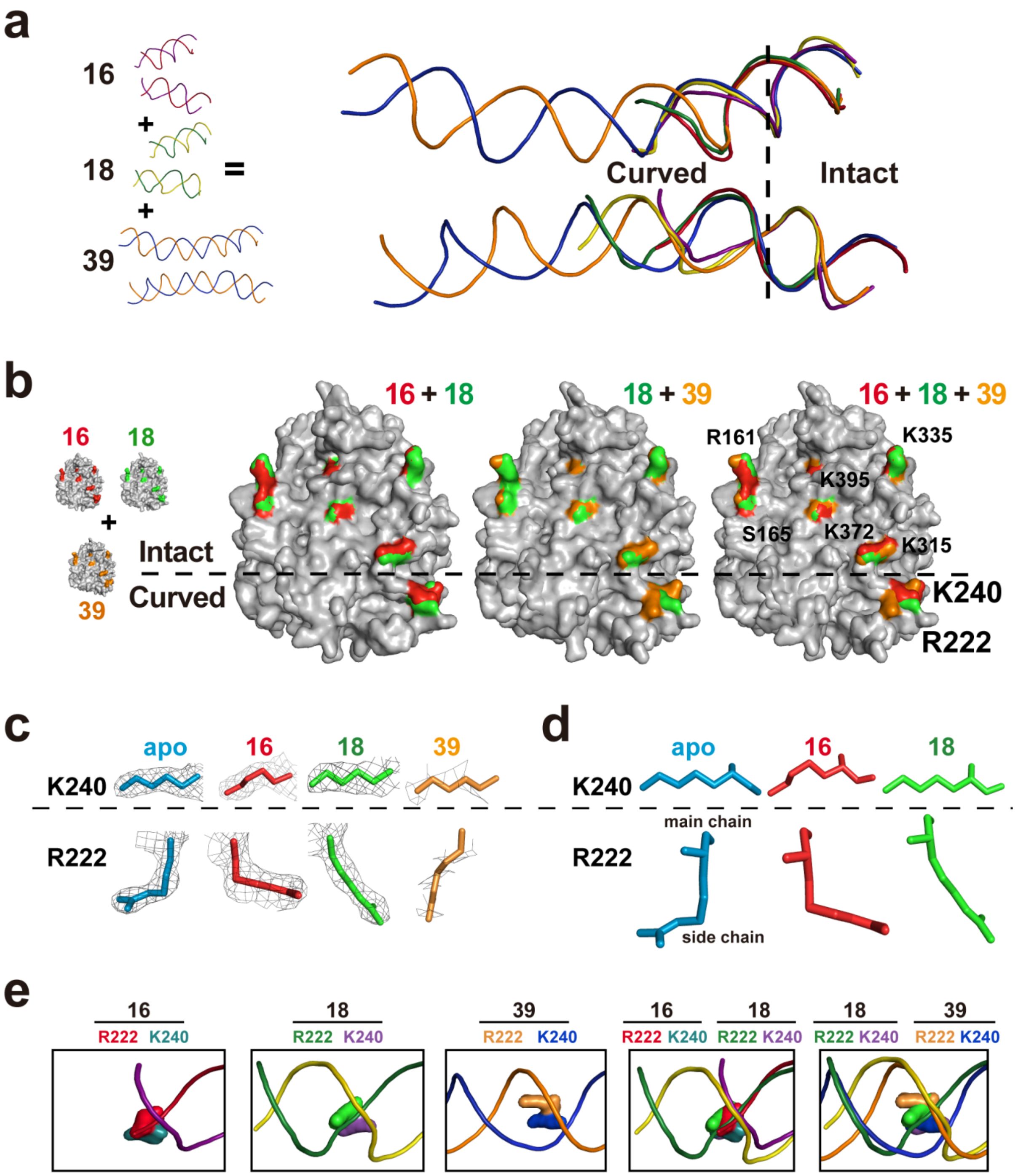

Supplementary Fig. 10 Structural analyses of R222 in the detection of DNA-flexibility. a, DNA superposition of the 16-bp (purple and red), 18-bp (green and yellow) and 39-bp (blue and orange) atomic models. Intact: the intact DNA zone. Curved: the curved DNA zone. 16-bp complex: Kbss: red; sc–Kbss: purple. 18-bp complex: Kbss: green; sc–Kbss: yellow. 39-bp complex: Kbss: blue; sc–Kbss: orange. **b,** Conserved residue superposition of the 16 (red), 18 (green) and 39 (orange) atomic models. Intact: the intact DNA zone. Curved: the curved DNA zone. **c,** The electronic densities of R222 and K240 side chains in the apo (blue), 16 (red), 18 (green) and 39 (orange) atomic models. Side chains are shown by stick representations. **d,** The main and side chains of R222 and K240 in the apo (blue), 16 (red) and 18 (green) atomic models. Main and side chains are shown by stick representations. Due to the resolution limitation shown in (**c**), the electronic densities of R222 and K240 in 39 atomic model were not applied for the further quantitative analyses of side chain. **e,** Superposition of the noted atomic models. The DNA main chains and residues are shown by ribbon and surface representations, respectively.

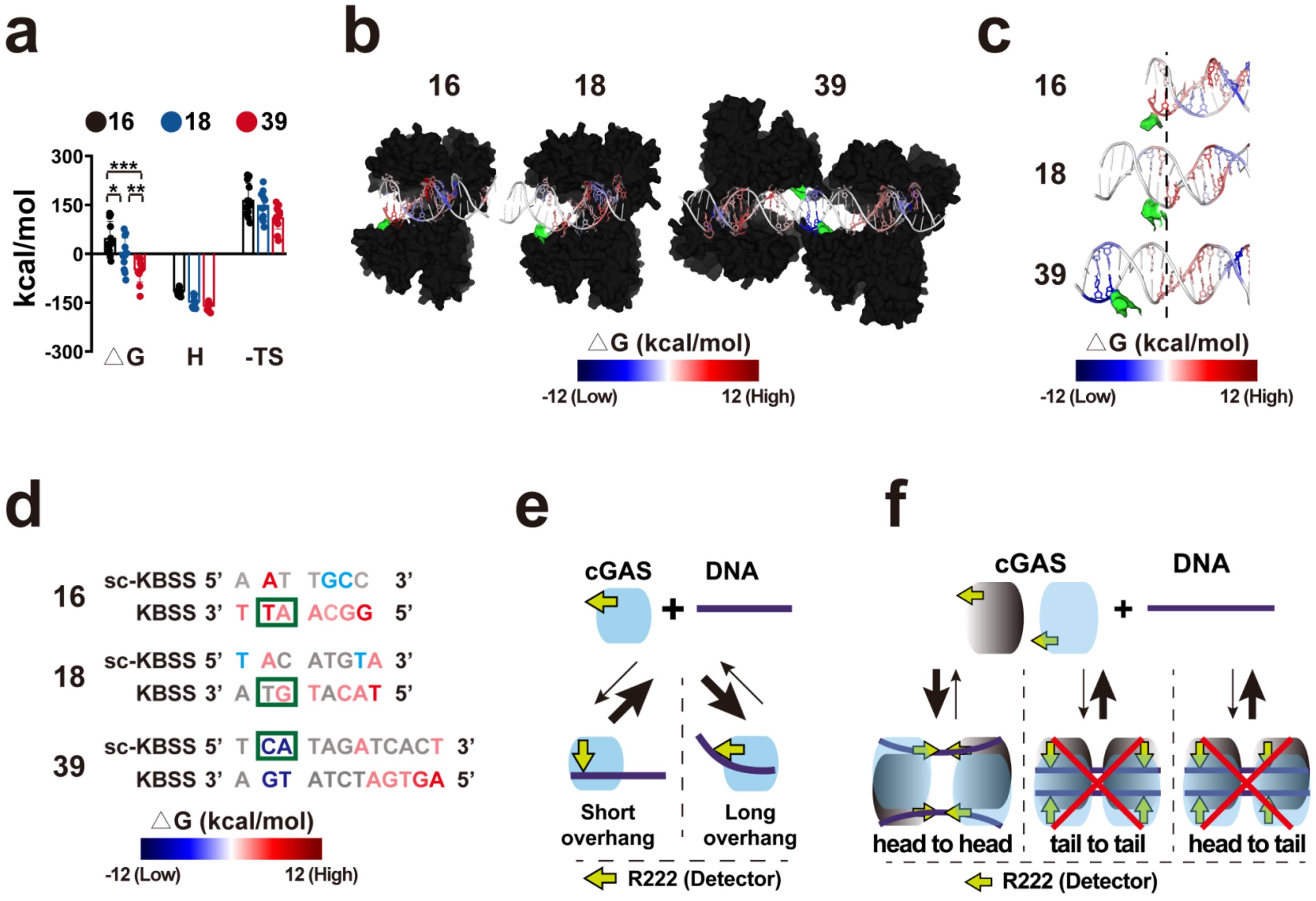

Supplementary Fig. 11 The free energy of cGAS–DNA binding in the DNA–cGAS complexes. a, Free energy (△G) of protein–DNA binding in 16-bp, 18-bp and 39-bp DNA–cGAS atomic models were assessed by gmx_MMPBSA (version 1.5.6). 16-bp and 18-bp DNA bind cGAS to form $cGAS_2$–DNA_2 complex, while 39-bp DNA binds cGAS to form $cGAS_4$–DNA_2 complex. To normalize this structural difference, the values of △G, H and –TS from 39-bp DNA–cGAS complex were divided by 2. Mean ± SD. n = 12 technical replicates. **b,c,** Free energy (△G) of each DNA base in 16-bp, 18-bp and 39-bp DNA–cGAS complexes. The △G for each DNA base were calculated by gmx_MMPBSA using the default parameters. cGAS and DNA are shown by surface and ribbon representations, respectively. DNA bases were colored according to their △G values. The cGAS proteins were hidden in (**c**). Green: R222. Red: △G high; Blue: △G low. **d,** The △G of R222-binding DNA base in 16-bp, 18-bp and 39-bp DNA–cGAS complexes. DNA bases were colored according to their △G values. R222-binding DNA bases were highlighted with green rectangle. Kbss: K240-binding single-strand; sc–Kbss: complementary to K240-binding single-strand. **e,** Schematic showing that cGAS–DNA complex with a longer overhang DNA at R222 might be more stable. Given that DNA-length considerably elevates the DNA-flexibility, it might be more energetically favorable to curve a long overhang DNA at R222 than a short overhang DNA. **f,** Schematic showing the head-to-head structure of the $cGAS_4$–DNA_2 complex. If R222 end of cGAS is taken as head and the other end is taken as tail, the two cGAS dimers in 39-bp DNA–cGAS complex are arranged in a head-to-head (but not head-to-tail or tail-to-tail) orientation to form the $cGAS_4$–DNA_2 structure. Notably, two cGAS dimers arranged in head-to-head orientation can result in a longer overhang DNA at R222, suggesting that cGAS–DNA complex with a longer overhang DNA at R222 might be energetically favorable. Source data are provided as a Source Data file.

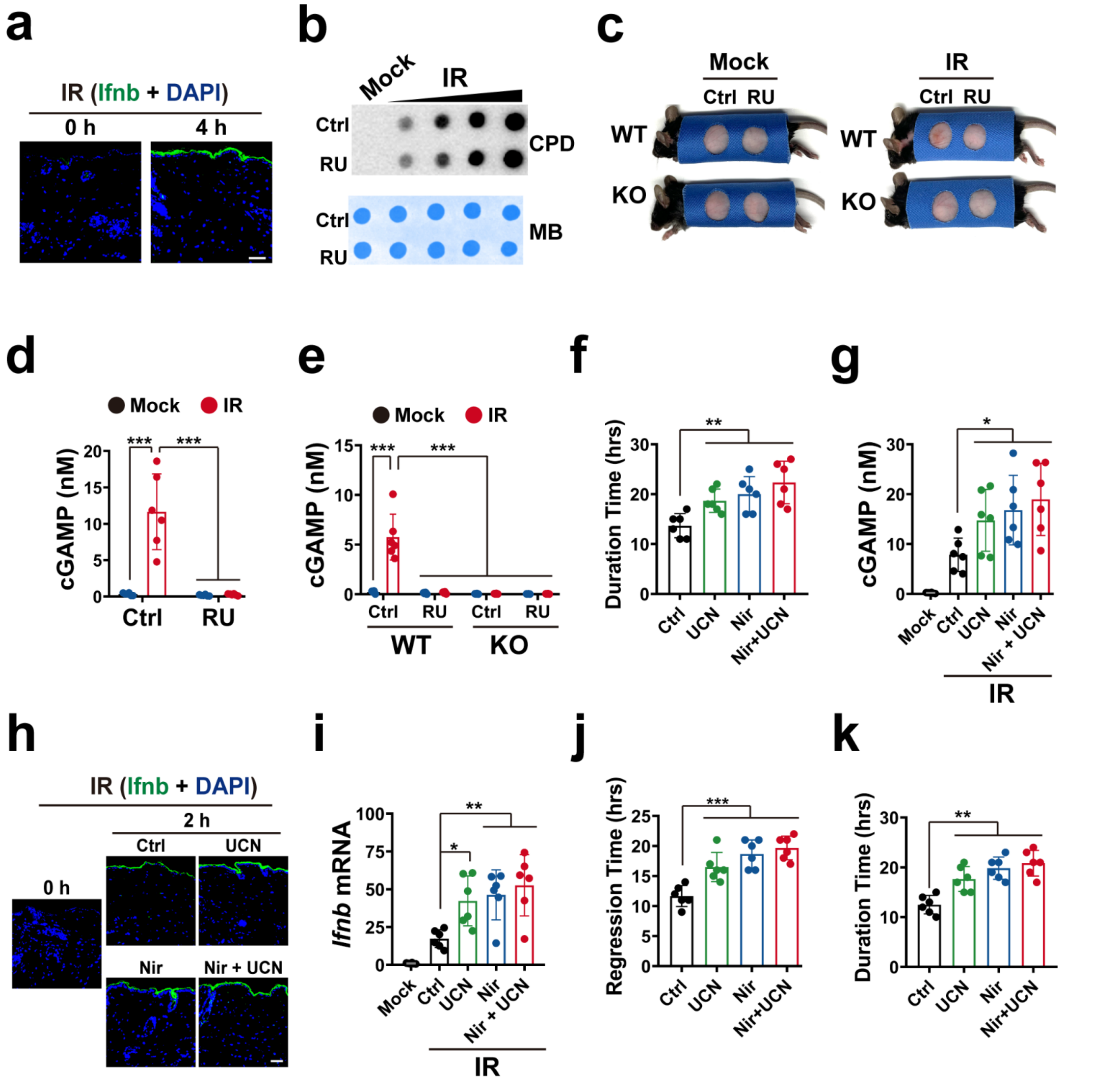

Supplementary Fig. 12 Flexsurveillance model suggests that low dose IR might activate cGAS-mediated AIS via repairable DNA. a, Representative IF images of the nude mice at noted times after IR. Blue: Dapi; Green: Ifnb. Scale bar = 100 μm. **b,** Dot blot analyses of cyclobutane pyrimidine dimer (CPD) in the genomic DNAs from the Ctrl or RU.521-pretreated mice at 20 min after IR. **c,** Representative images of the cGAS wild type (WT) and knock out (KO) C57BL/6 mice at 4 h after 400 mJ/cm^2 IR. **d,** cGAMP levels in the skin samples from the RU.521-pretreated nude mice at 1 h after 300 mJ/cm^2 IR. In this study, all data are presented as mean values ± standard deviations (SDs), and error bars indicate SDs. Mean ± SD. n = 6 mice. **e,** cGAMP levels in the skin samples from the RU.521-pretreated cGAS WT and KO mice at 1 h after IR. Mean ± SD. n = 6 mice. **f,** Duration times of erythema in the mice described in Fig. 8h. Mean ± SD. n = 6 mice. **g,** cGAMP levels in the skin samples from the inhibitor-pretreated mice at 1 h after 300 mJ/cm^2 IR. Samples from the mice at 0 h after IR were used as mock. Mean ± SD. n = 6 mice. **h,** Representative IF images of the mice treated as described in Fig. 8h at noted times after IR. Blue: Dapi; Green: Ifnb. Scale bar = 100 μm. **i,** Ifnb levels in the skin samples from the mice treated as described in Fig. 8h at 2 h after IR. Samples from the mice at 0 h after IR were used as mock. Mean ± SD. n = 6 mice. **j,k,** The regression (**j**) and duration (**k**) times of erythema in the mice treated as described in Fig. 8h on the two days after the first round treatment. Mean ± SD. n = 6 mice. Source data are provided as a Source Data file.

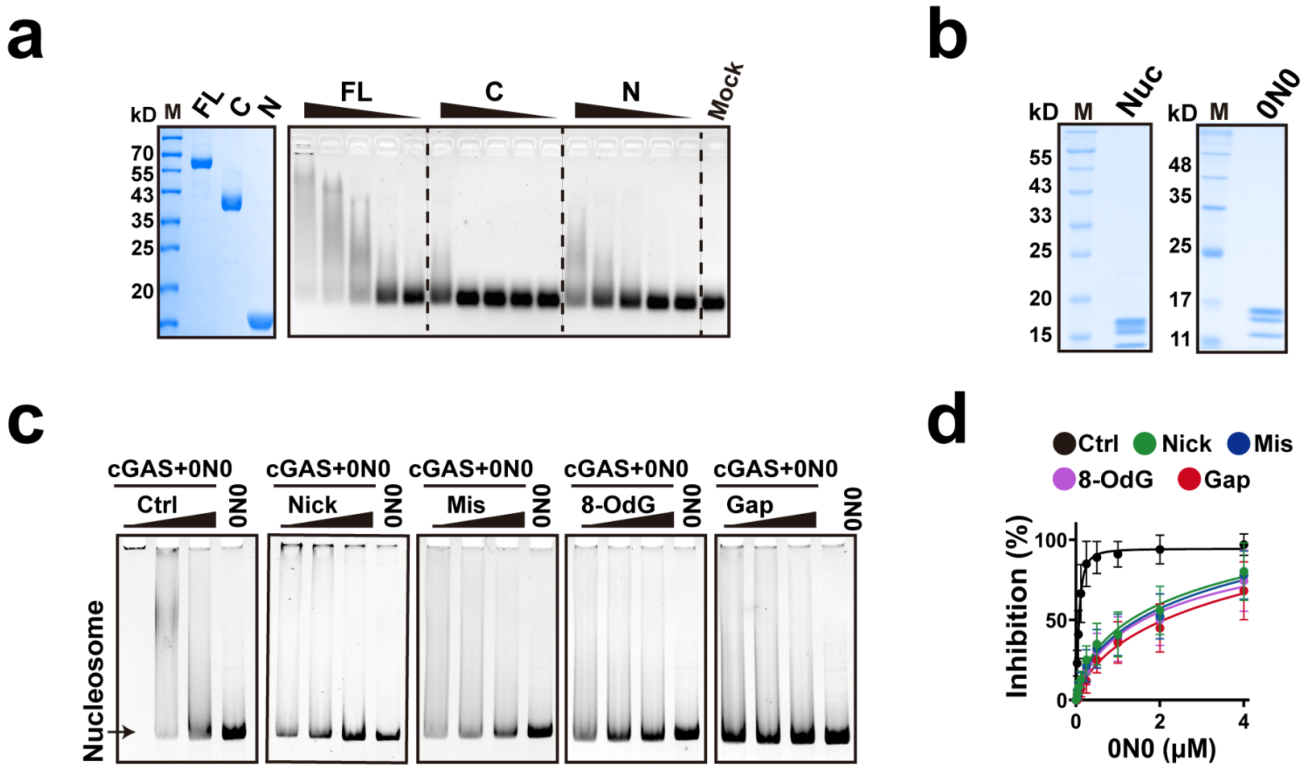

Supplementary Fig. 13 Damages elevate the potential of nucleosomal DNA to bind and activate cGAS. a, Left: Coomassie staining of in vitro purified human full length (FL), truncated C-terminal (C) and N-terminal (N) hcGAS proteins. Right: EMSA analyses of 50 nM 44-bp ISD binding to serial dilutions of noted cGAS proteins. **b,** Coomassie staining of nucleosome without DNA (Nuc) and nucleosome with DNA (0N0). **c,** EMSA assays of 25 nM fluorescently labelled nucleosome (0N0) binding to 200 nM cGAS in the presence of serial dilutions of 44-bp noted ISD. 25 nM Cy3-labelled nucleosome (0N0) and 200 nM cGAS were mixed with serial dilutions of ISD that was not fluorescently labelled. After incubation, the mixtures were resolved on gel. **d,** Inhibition of 50 nM cGAS by nucleosome (0N0) in the presence of 100 nM noted DNAs. The nucleosome (0N0) IC_{50} (half maximal inhibitory concentration) of cGAS for Ctrl: 75.42 ± 20.6 nM, Nick: 1655.0 ± 412.5 nM, Mismatch: 1734.1 ± 485.6 nM, 8-OdG: 2001.3 ± 516.4 nM and Gap: 2102.6 ± 585.1 nM. Mean ± SD. n = 3 independent experiments. Source data are provided as a Source Data file.

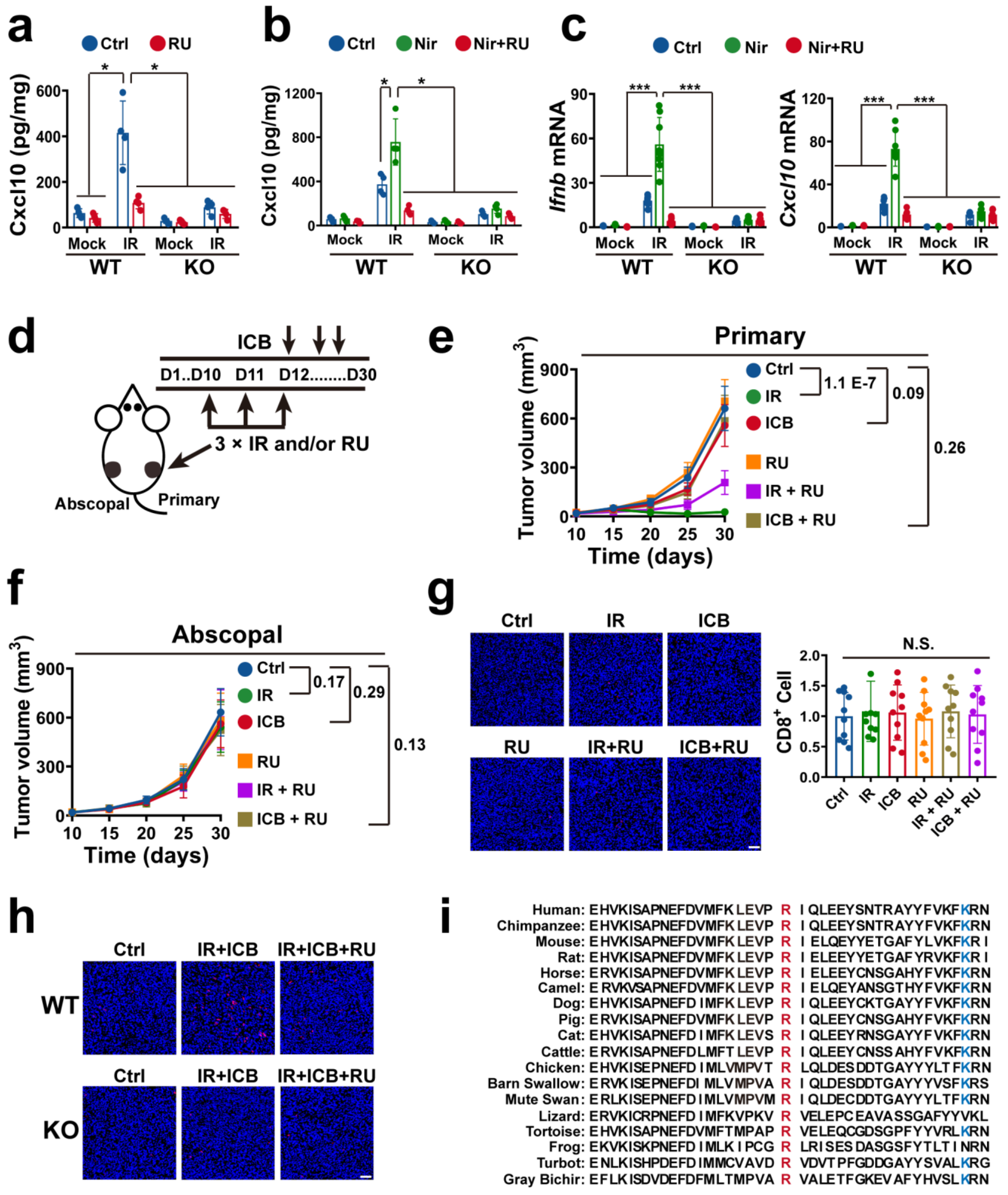

Supplementary Fig. 14 Loss of cGAS-mediated daily AIS impairs the radiotherapy efficiency. a, Cxcl10 protein levels in the tumors at 3 h after single focal 8 Gy IR. Mean ± SD. n = 4 tumors from 4 mice. In this study, all data are presented as mean values ± standard deviations (SDs), and error bars indicate SDs. **b,** Cxcl10 protein levels in the inhibitor-pretreated tumors at 3 h after single 8 Gy IR. Mean ± SD. Mice: n = 4; Tumor: n = 4. **c,** Ifnb (left) and Cxcl10 (right) mRNA levels at 4 h after IR on the second day in the mice treated as described in Fig. 10c on two consecutive days. Mean ± SD. MRNA: n = 8 tumors from 4 mice. **d,** Schematic showing primary (right flank) tumors treated by three focal fractions of 8 Gy IR (on D10, D11 and D12). **e,f,** Primary (right flank) (**e**) and abscopal (left flank) (**f**) tumor volumes in the RU.521-pretreated WT and KO mice treated as described in (**d**). Mean ± SD. n = 10 mice. **g,** Left: Representative images showing CD8+ T cells infiltrating the abscopal tumors in the mice described in (**f**). Blue: Dapi; Red: anti-CD8 antibody. Scale bar = 100 µm. Right: CD8+ T cells in the abscopal tumors described in (**f**). Mean ± SD. n = 10 mice (tumors). **h,** Representative images showing CD8+ T cells infiltrating the abscopal tumors in the mice described in Fig. 10i. Blue: Dapi; Red: anti-CD8 antibody. Scale bar = 100 µm. **i,** Multiple sequence alignment showing the conservation of the detector mouse R222. Mouse R222: red; K240: blue. Source data are provided as a Source Data file.
