# Supplementary material for: DNA mechanical flexibility controls DNA potential to activate cGAS-mediated immune surveillance
Source: Nat Commun. 2022 Nov 19;13:7107. doi: 10.1038/s41467-022-34858-6 (PMC9675814; doi:10.1038/s41467-022-34858-6)
Supplement: Supplementary file 2 — Description of additional Supplementary File [file 41467_2022_34858_MOESM2_ESM.pdf]

Descriptions of Additional Supplementary Files

Supplementary Data 1 describes the sequences of oligonucleotides used in this study.
