# Supplementary material for: DNA mechanical flexibility controls DNA potential to activate cGAS-mediated immune surveillance
Source: Nat Commun. 2022 Nov 19;13:7107. doi: 10.1038/s41467-022-34858-6 (PMC9675814; doi:10.1038/s41467-022-34858-6)
Supplement: Supplementary file 4 — Reporting Summary [file 41467_2022_34858_MOESM4_ESM.pdf]

nature portfolio

Corresponding author(s): Qingkai Yang
Last updated by author(s): Oct 30, 2022

Reporting Summary

Nature Portfolio wishes to improve the reproducibility of the work that we publish. This form provides structure for consistency and transparency in reporting. For further information on Nature Portfolio policies, see our Editorial Policies and the Editorial Policy Checklist.

Statistics

For all statistical analyses, confirm that the following items are present in the figure legend, table legend, main text, or Methods section.

n/a	Confirmed	
☐	☒	The exact sample size (*n*) for each experimental group/condition, given as a discrete number and unit of measurement
☐	☒	A statement on whether measurements were taken from distinct samples or whether the same sample was measured repeatedly
☐	☒	The statistical test(s) used AND whether they are one- or two-sided<br>*Only common tests should be described solely by name; describe more complex techniques in the Methods section.*
☒	☐	A description of all covariates tested
☐	☒	A description of any assumptions or corrections, such as tests of normality and adjustment for multiple comparisons
☐	☒	A full description of the statistical parameters including central tendency (e.g. means) or other basic estimates (e.g. regression coefficient) AND variation (e.g. standard deviation) or associated estimates of uncertainty (e.g. confidence intervals)
☒	☐	For null hypothesis testing, the test statistic (e.g. *F*, *t*, *r*) with confidence intervals, effect sizes, degrees of freedom and *P* value noted<br>*Give P values as exact values whenever suitable.*
☒	☐	For Bayesian analysis, information on the choice of priors and Markov chain Monte Carlo settings
☒	☐	For hierarchical and complex designs, identification of the appropriate level for tests and full reporting of outcomes
☐	☒	Estimates of effect sizes (e.g. Cohen's *d*, Pearson's *r*), indicating how they were calculated

Our web collection on statistics for biologists contains articles on many of the points above.

Software and code

Policy information about availability of computer code

Data collection	The Analyst software for Windows (Applied Biosystems, Darmstadt, Germany) (version 1.6.2) was used for was used for data acquisition and MS peak area quantification.
Data analysis	The atomic models were generated computationally using PyMOL Molecular Graphic Systems (version 2.5.2). Conformational energetic changes of R as a free anima acid are calculated by Hyperchem (version 9.01). The free energy of protein–DNA binding was assessed using gmx_MMPBSA (version 1.5.6). Statistical data were analyzed by GraphPad Prism8.

For manuscripts utilizing custom algorithms or software that are central to the research but not yet described in published literature, software must be made available to editors and reviewers. We strongly encourage code deposition in a community repository (e.g. GitHub). See the Nature Portfolio guidelines for submitting code & software for further information.

Data

Policy information about availability of data

All manuscripts must include a data availability statement. This statement should provide the following information, where applicable:

- Accession codes, unique identifiers, or web links for publicly available datasets
- A description of any restrictions on data availability
- For clinical datasets or third party data, please ensure that the statement adheres to our policy

A reporting summary for this article is available as Supplementary Information file. The data supporting the findings of this study are available within the article and its Supplementary Figures. The source data underlying Fig. 2–5, Fig. 8–10, Supplementary Fig. 1–9 and Supplementary Fig. 11–14 are provided as a Source Data file.

The structural data of apo-(Protein Data Bank (PDB): 4K8V [https://www.rcsb.org/structure/4K8V]), 16-bp (PDB: [https://www.rcsb.org/structure/4K96]), 18-bp (PDB: 4LEZ [https://www.rcsb.org/structure/4LEZ]) and 39-bp (PDB: 5N6I [https://www.rcsb.org/structure/5N6I]) DNA–cGAS complexes were derived from the Protein Data Bank database (https://www.rcsb.org). More details on datasets and protocols that support the findings of this study will be available upon request. Source data are provided with this paper.

Human research participants

Policy information about studies involving human research participants and Sex and Gender in Research.

Reporting on sex and gender	N/A
Population characteristics	N/A
Recruitment	N/A
Ethics oversight	N/A

Note that full information on the approval of the study protocol must also be provided in the manuscript.

Field-specific reporting

Please select the one below that is the best fit for your research. If you are not sure, read the appropriate sections before making your selection.

[x] Life sciences [ ] Behavioural & social sciences [ ] Ecological, evolutionary & environmental sciences

For a reference copy of the document with all sections, see nature.com/documents/nr-reporting-summary-flat.pdf

Life sciences study design

All studies must disclose on these points even when the disclosure is negative.

Sample size	In general, no calculations were done to determine sample size. Sample size was determined based on standards for experimental in vitro, cellular and animal studies, leading to a minimum of n = 3 biological replicates with sufficient reproducibility. Hence, n = 6 was used for the most experiments.
Data exclusions	No data was excluded from analysis. For the MS analysis of cGAMP, quantification was based on the parent ion to product ions: m/z 675 to 136, because m/z 136 is the most notable and repeatable peak.
Replication	All experiments were replicated at least three times, and all attempts at replication were successful.
Randomization	We used simple random sampling.
Blinding	Yes. The treatment, data collection and analysis were carried out by different investigators.

Reporting for specific materials, systems and methods

We require information from authors about some types of materials, experimental systems and methods used in many studies. Here, indicate whether each material, system or method listed is relevant to your study. If you are not sure if a list item applies to your research, read the appropriate section before selecting a response.

Materials & experimental systems

n/a	Involved in the study	
[ ]	[x]	Antibodies
[ ]	[x]	Eukaryotic cell lines
[x]	[ ]	Palaeontology and archaeology
[ ]	[x]	Animals and other organisms
[x]	[ ]	Clinical data
[x]	[ ]	Dual use research of concern

Methods

n/a	Involved in the study	
[x]	[ ]	ChIP-seq
[x]	[ ]	Flow cytometry
[x]	[ ]	MRI-based neuroimaging

Antibodies

Antibodies used	Anti-Ifnb1 (D2J1D) antibody, Cell Signaling Technology (Cat. number: 97450);

	Anti-Actin antibody, Cell Signaling Technology (Cat. number: 4967);<br>Anti-cGAS (D3O8O) antibody, Cell Signaling Technology (Cat. number: 31659);<br>Anti-BrdU antibody, Cell Signaling Technology (Cat. number: 5292);<br>Anti-cyclobutane pyrimidine dimers (CPDs) antibody, Cosmo Bio (Cat. number: CAC-NM-DND-001);<br>Anti-Ctla4 (mAb Clone: 9H10) antibody, Bio X Cell (Cat. number: BE0131);<br>Anti-CD8 (EPR21769) antibody, abcam (Cat. number: ab217344);
Validation	Anti-Ifnb1 (D2J1D) antibody, Cell Signaling Technology (Cat. number: 97450)<br>Species: Mouse<br>Application: Western Blot; Flow Cytometry<br>Citation: PMID: 31959883; PMID: 33672392<br><br>Anti-Actin antibody, Cell Signaling Technology (Cat. number: 3700)<br>Species: Human, Mouse, Rat<br>Application: Western Blot; Flow Cytometry; Immunohistochemistry; Immunofluorescence;<br>Citation: PMID: 35896539; PMID: 35869048<br><br>Anti-cGAS antibody, Cell Signaling Technology (Cat. number: 31659)<br>Species: Mouse<br>Application: Western Blot; Immunoprecipitation<br>Citation: PMID: 34739325; PMID: 35322803<br><br>Anti-BrdU antibody, Cell Signaling Technology (Cat. number: 5292)<br>Species: ALL<br>Application: Immunohistochemistry; Immunofluorescence; Flow Cytometry<br>Citation: PMID: 34995493; PMID: 33436545<br><br>Anti-cyclobutane pyrimidine dimers (CPDs) antibody, Cosmo Bio (Cat. number: CAC-NM-DND-001)<br>Species: ALL<br>Application: Dot Blot; Immunohistochemistry; Flow Cytometry; Western Blot<br>Citation: PMID: 17236820; PMID: 15882621<br><br>Anti-CD8 antibody, abcam (Cat. number: ab217344)<br>Species: Mouse<br>Application: Western Blot; Immunohistochemistry; Flow Cytometry<br>Citation: PMID: 33420008; PMID: 33514544<br><br>Anti-Ctla4 (mAb Clone: 9H10) antibody, Bio X Cell (Cat. number: BE0131)<br>Species: Mouse<br>Application: in vivo CTLA-4 neutralization; in vitro CTLA-4 neutralization; Western Blot<br>Citation: PMID: 27667683; PMID: 29339377

Eukaryotic cell lines

Policy information about cell lines and Sex and Gender in Research

Cell line source(s)	MC38 and THP-1 cells were from ATCC.
Authentication	MC38 and THP-1 cell lines were subjected to short tandem repeat (str) authentication to confirm genomic stability. The cell-handling procedures were approved by the Ethics Committee of DaLian Medical University.
Mycoplasma contamination	All cell lines were tested negative for Mycoplasma contamination.
Commonly misidentified lines (See ICLAC register)	No misidentified line was used.

Animals and other research organisms

Policy information about studies involving animals; ARRIVE guidelines recommended for reporting animal research, and Sex and Gender in Research

Laboratory animals	C57BL/6 female mice, age 5–8 weeks old. All mice were housed in a specific pathogen free facility at 22 ± 2ºC under a cycle of 12 h light (7:00 am light on) and 12 h dark (7:00 pm light off). Once the longest diameter of tumor was above 1.5 cm, mice were sacrificed according to the handling procedures of the Animal Care and Use Committee.
Wild animals	No wild animals were used in the study.
Reporting on sex	Findings should not be applied to just one sex. Because male mice might be more variable than female mice who all just get along in a cage, female mice were used for the tumor challenge and treatment.

Field-collected samples	None collected.
Ethics oversight	The animal handling procedures were approved by the Animal Care and Use Committee of DaLian Medical University.

Note that full information on the approval of the study protocol must also be provided in the manuscript.
